# Can global or national disability weights represent provincial level?

**DOI:** 10.1186/s12889-022-14347-7

**Published:** 2023-03-10

**Authors:** Qin He, Maigeng Zhou, Peng Yin, Bo Jiang, Jinlei Qi, Yunning Liu, Jiangmei Liu, Jinling You, Yuchang Zhou, Zheng Long, Xiuya Xing, Qianyao Cheng, Yeji Chen, Huadong Wang, Zhirong Liu

**Affiliations:** 1 Anhui Provincial Center for Disease Control and Prevention, Shushan District, Hefei, 230601 China; 2 National Center for Chronic and Non-Communicable Disease Control and Prevention, Chinese Center for Disease Control and Prevention, Xicheng District, Beijing, 100050 China

**Keywords:** Disability weight, Health state, Paired comparison, Probit regression model, Cross-cultural comparisons

## Abstract

**Background:**

A universal set of disability weights(DWs) is mainly based on the survey of North America, Australia and Europe, whereas the participants in Asia was limited. The debate hasn’t yet settled whether a universal DW is desirable or useful.The focus of the debate is its representativenes-s.After all, the DWs come from people's subjective evaluation of pain, and they may vary according to cultural background.The differences of the DWs could have implications for the magnitude or ranking of disease burdens.The DWs of Anhui Province has not been completely presented.This paper aims to obtain the DWs suitable for the general population of Anhui Province of China, and attempts to explore the differences between different DWs by comparing the DWs with the similar-cultural background and the DWs with cross-cultural background.

**Methods:**

A web-based survey was conducted to estimate the DWs for 206 health states of Anhui province in 2020. Paired comparison (PC) data were analyzed and anchored by probit regression and fitting loess model. We compared the DWs in Anhui with other provinces in China and those in Global burden of disease (GBD) and Japan.

**Results:**

Compared with Anhui province, the proportion of health states which showed 2 times or more differences ranged from 1.94% (Henan) to 11.17% (Sichuan) in China and domestic provinces. It was 19.88% in Japan and 21.51% in GBD 2013 respectively. In Asian countries or regions, most of the health states with top 15 DWs belonged to the category of mental, behavioral, and substance use disorders. But in GBD, most were infectious diseases and cancer. The differences of DWs in neighboring provinces were smaller than other geographically distant provinces or countries.

**Conclusion:**

PC responses were largely consistent across very distinct settings,but the exceptions do need to be faced squarely.The differences of DWs among similar-cultural regions were smaller than cross-cultural regions. There is an urgent need for relevant gold standards.

**Supplementary Information:**

The online version contains supplementary material available at 10.1186/s12889-022-14347-7.

## Background

In terms of resource allocation, impact monitoring of medical reform, effect evaluation of intervention measures and determination of health priorities, the burden of disease is an important reference [1–4]. Disability-adjusted life years (DALYs)is a generally recognized measure of disease burden, which comprehensively considers the impact of death and disability on health [1, 5, 6]. The DWs are an important parameter for calculating DALYs, and are used to quantify health levels associated with non fatal outcomes. The DWs range from 0(equivalent to full health) to 1(equivalent to death). A universal set of global DW was estimated in GBD study. As an important parameter, the debate hasn’t yet settled whether a universal set of DWs is desirable or useful [5–10], although many improvements have been made since it was first proposed. The global DWs are mainly based on the survey of North America, Australia and Europe [2, 3, 5, 7, 11, 12]. However, the DWs come from people's subjective evaluation of pain. People's perception of pain will be different in different countries or regions due to differences in cultural background [10–12]. The differences of the DWs could have substantial implications for the magnitude or ranking of disease burdens [12]. Korea [13–15], Japan [16] and China [17] have successively published their DWs. China is a populous country with a vast territory, and there are similar-cultural backgrounds among domestic provinces, but there are still some differences. Anhui’s data hasn’t been fully presented in Chinese study, this paper aims to obtain the DWs suitable for the general population of Anhui Province of China, and attempts to explore the differences between different DWs by comparing the DWs with the similar-cultural background and the DWs with cross-cultural background.

## Methods

### Study design and participants

A web-based survey of permanent residents aged 18–69 in Anhui province in 2020 was conducted through a self-administered questionnaire. The exclusion criteria for the participants were as follows: residents living in functional areas, such as work sheds, military, student dormitories, nursing homes; pregnant women; patients with mental disorders; uncooperative participants(??).

The questionnaire included a fixed personal demographics information and a PC which include 18 questions. Each question was composed of two options of different health states randomly selected from 206 health states, participants chose the healthier through their subjective feelings. Questions 3, 10 and 16 were used for quality control, and their contents overlapped with other questions.

### Description of health states and sample size

Supplementary Table S1 showed lay descriptions for 206 health states of the present study which is mainly based on GBD study. Each pair was required to be drawn no less than 10 times to ensure the stability of parameters, the minimum sample size was 28,150 (15 valid health states pairs per questionnaire, 206 × 205 ÷ 15 × 10 = 28,150).

### Survey procedure and quality control

Participants filled in the questionnaire through the national or provincial two-dimensional code of electronic questionnaire identified by mobile phone, and each we-chat (a popular instant messenger app in China) account could only fill in the questionnaire once. The questionnaires with inconsistent answers for the quality control questions or too short response time were eliminated at the end of the process. The project areas will carry out more targeted promotion and control deviation according to the weekly feedback of the project team throughout the survey.

### Statistical analysis

PC data were used to draw heat map and evaluate the data quality. Probit regression was constructed through PC data to obtain the coefficients that needs to be anchored in the interval of 0–1. Taking the coefficients as the independent variable, and the DWs of corresponding 172 health states in GBD 2013 transformed by logit as the dependent variable, loess model was used for anchoring the DWs. Using the random concept of Monte Carlo, 100 random probit regression coefficients were generated from the mean value and standard error of regression coefficients in each health state, so as to determine the 95% uncertainty interval (UI) of DW in each health state. Pearson correlation analysis was used to examine the correlation between the DWs of Anhui Province and that of other studies, P-values below 0.01 were deemed statistically significant. All of the analyses were performed with R (version 4.1.2).

## Results

### Analysis of DWs in anhui province

**Fig. 1:**
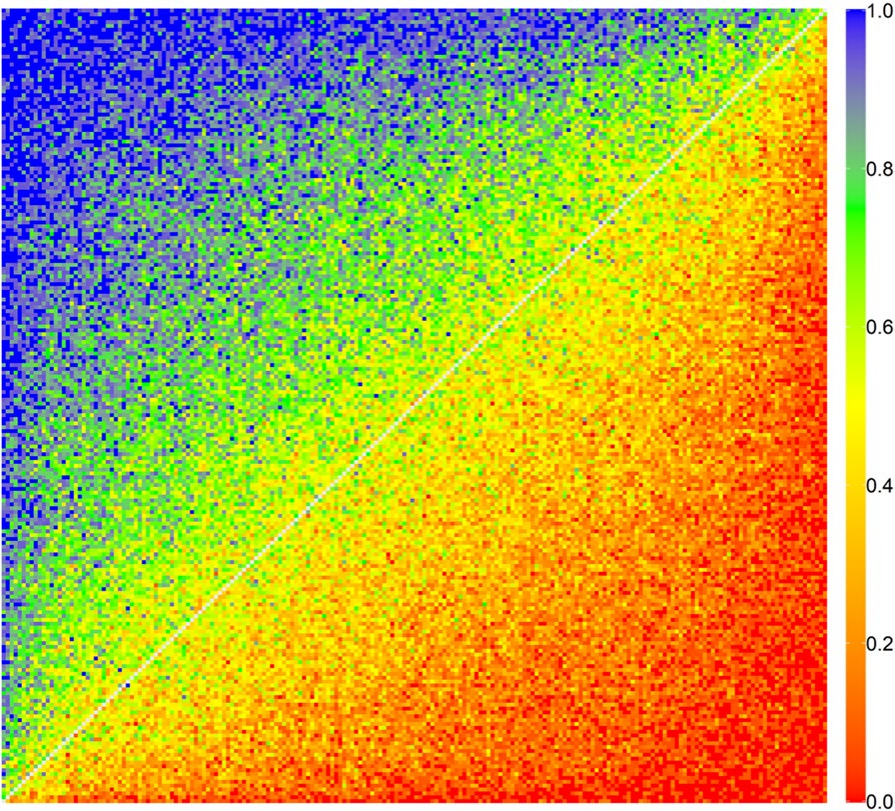
Response probabilities for paired comparisons in the web survey of Anhui province. Each cell indicated the response probability for one pair of states. The colors correspond to the probability that the first health state in PC was chosen as the healthier outcome

39,446 valid questionnaires were collected in Anhui province, which including 6258 questionnaires generated through the national two-dimensional code. Supplementary Table S2 showed the basic characteristics of participants and the comparison with the provincial population characteristics. Female or the participants aged 30–49 accounted for about 60%. Less than 10% were people with low education level. Except for the educational level, there was no significant difference between the sample population and the provincial population in terms of gender, age, ethnic and regional distribution(*p*-values > 0.01).The smooth color transition in the heat map showed that the data had small measurement error and good internal consistency (Fig. 1).

The DWs in Anhui province ranged from 0.003 (UI 0.002–0.008) for mild impairment of distance vision and mild anemia to 0.668 (UI 0.512–0.784) for severe heroin and other opioid dependence. DWs in different health states by descending order didn’t violate common sense, the only exception was the DW of cancer was lower than that of mild anemia. For the same states with different severity, only in 5 states such as tuberculosis with or without HIV infection, terminal phase with or without medication (for cancers and end-stage kidney or liver disease), varying degrees of hearing loss or distance vision and neck pain, the orders of DW didn’t increase with severity (Supplementary Table S3).

### Comparisons

This study involved 206 health states of 11 kinds of diseases, which is completely consistent with Chinese study [17] except for the serial number of health states, of which 172 were consistent with the GBD 2013 and 161 with Japanese study [16] (Supplementary Table S4). A more in-depth comparison will be made for the following aspects:

#### Comparison with other provinces in China

Supplementary Figure SF1 showed the DWs of Anhui province were highly correlated with the results of 8 provinces with provincial representativeness sample in China (r ≥ 0.985, *p* < 0.001). Compared with them, none of Anhui's DW was 2 times or more. In Table 1, the numbers of health states which showed 2 times or more differences compared with Anhui were 4 (1.94%) in neighboring Henan, 7 (3.40%) in Fujian and Liaoning, 10 (4.85%) in neighboring Jiangsu, 14 (6.80%) in neighboring Hubei, 17 (8.25%) in Yunnan, 20 (9.71%) in Hunan, 23 (11.17%) in Sichuan respectively. The DWs for mild impairment distance vision, mild anemia in other provinces except Henan were more than 3 times, the largest proportional difference was 4.33 times.

**Table 1.**
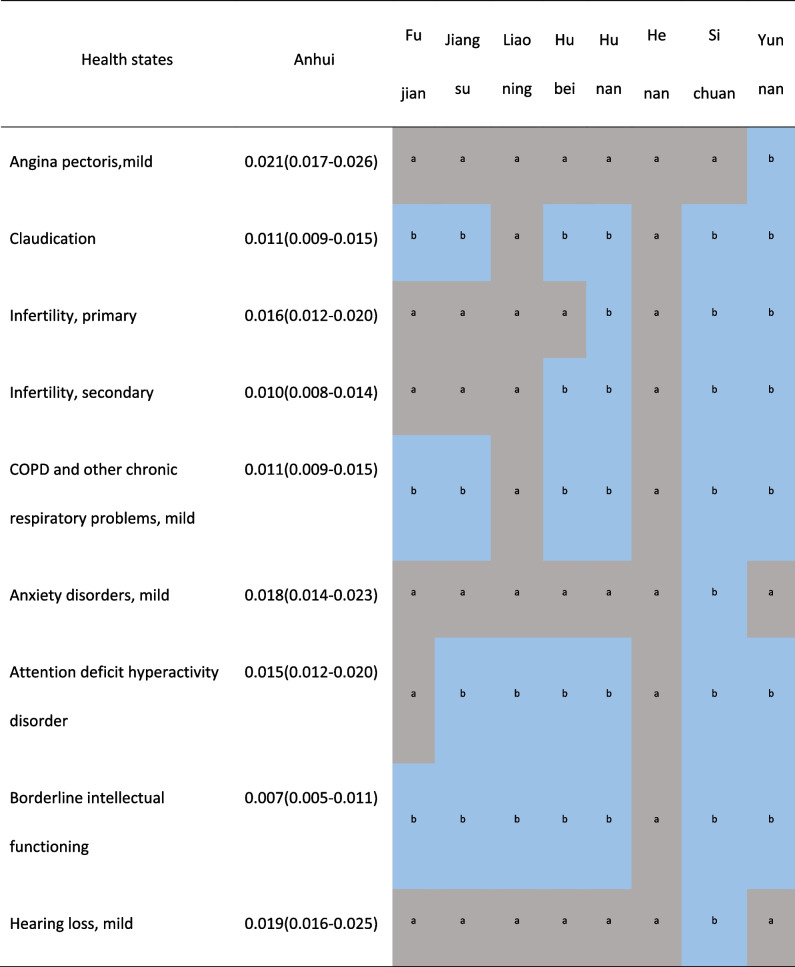

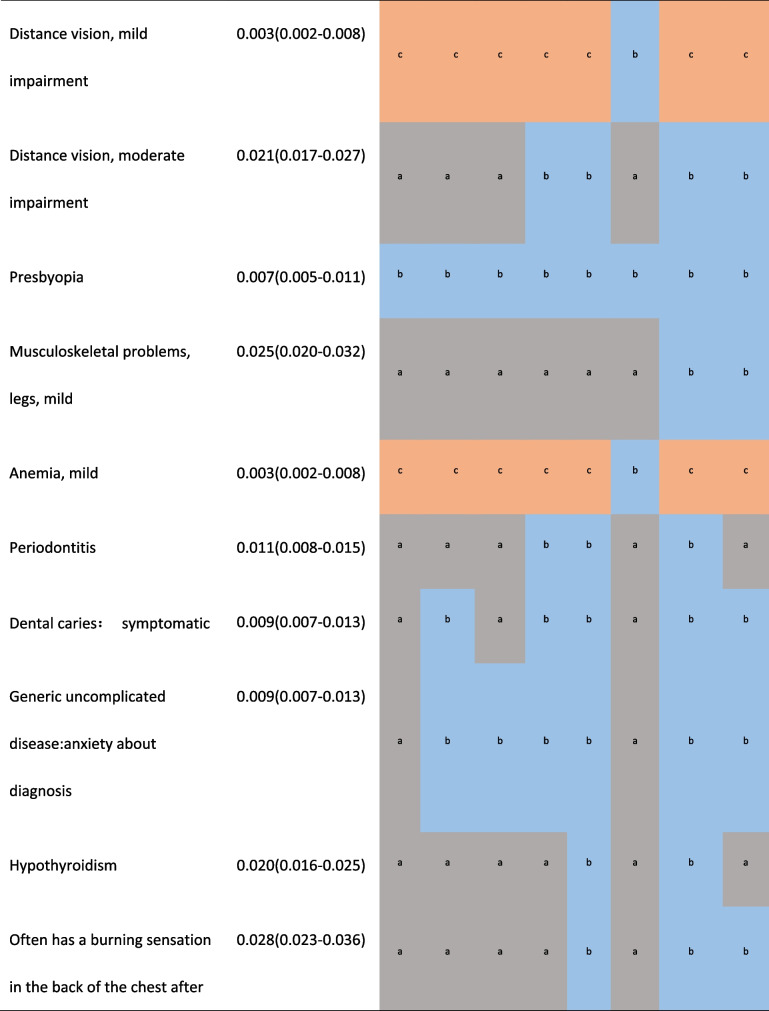

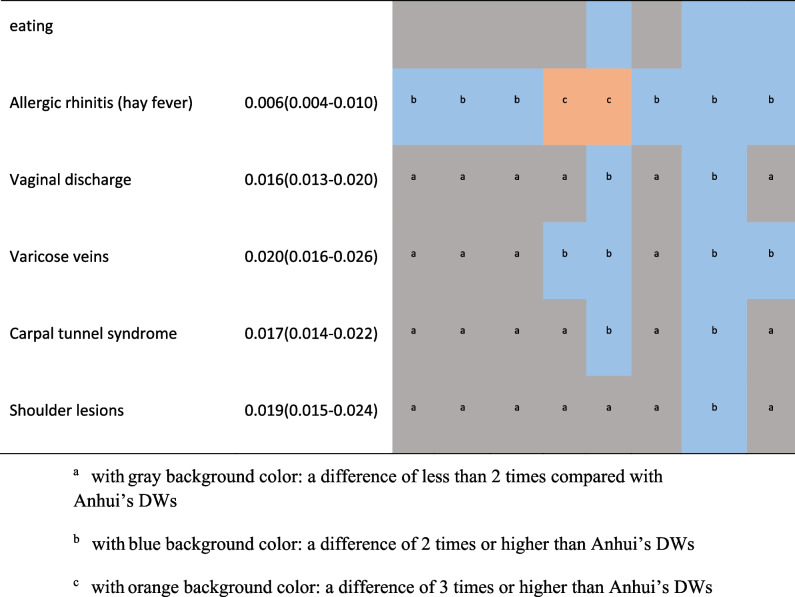
Comparison of the health states with 2 times or higher DW between Anhui and 8 provinces in China [17]

Ignoring the differences of their specific DWs and the ranking, most of the health states involving the top 15 and last 15 DWs overlapped among provinces. Nearly 56.67% of the top 15 DWs in these provinces belonged to the category of mental, behavioral, and substance use disorders. About 90% of the last 15 DWs belonged to other diseases. The DWs for mode rate to severe heroin and other opioid dependence ranked first in these provinces (Table 2).

**Table 2.**
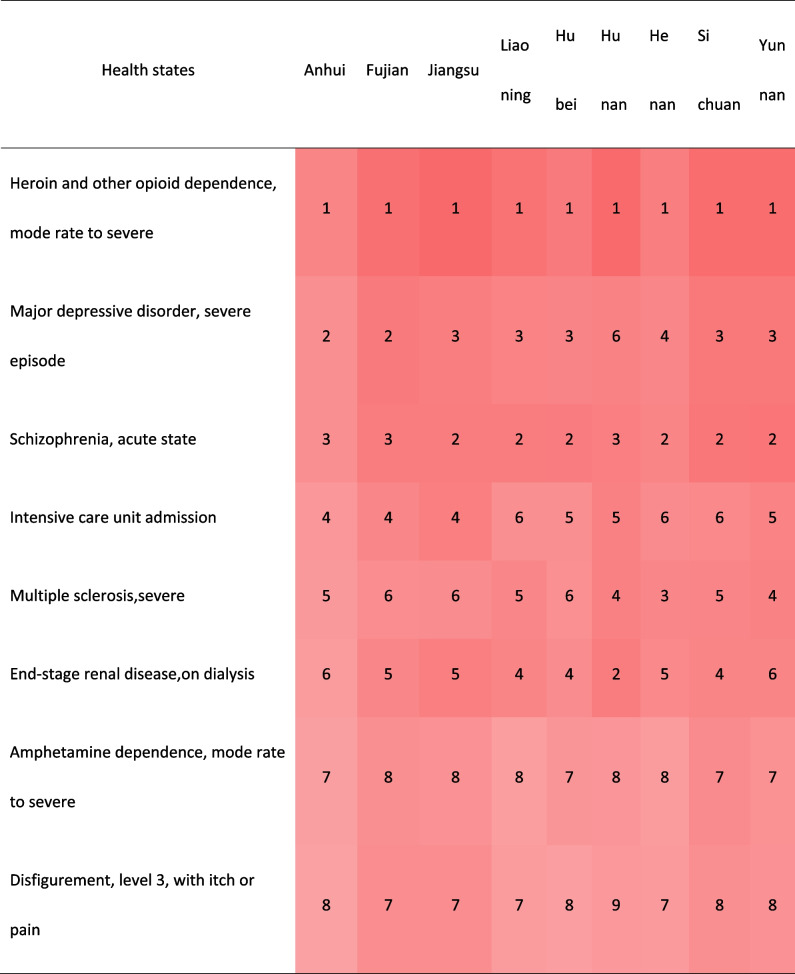

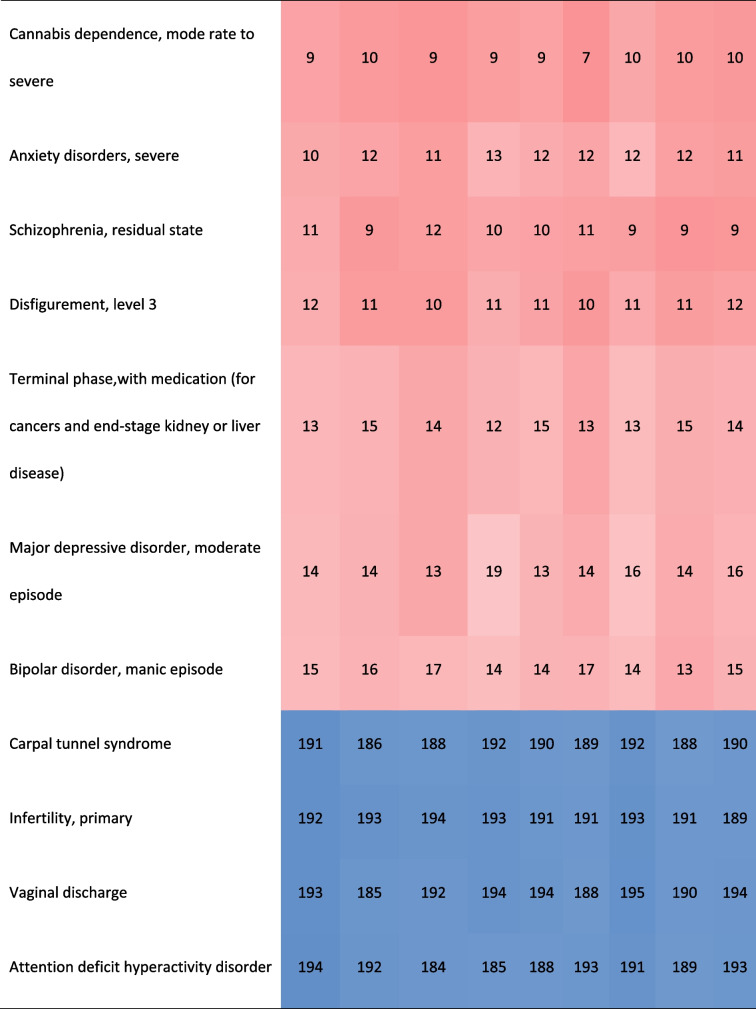

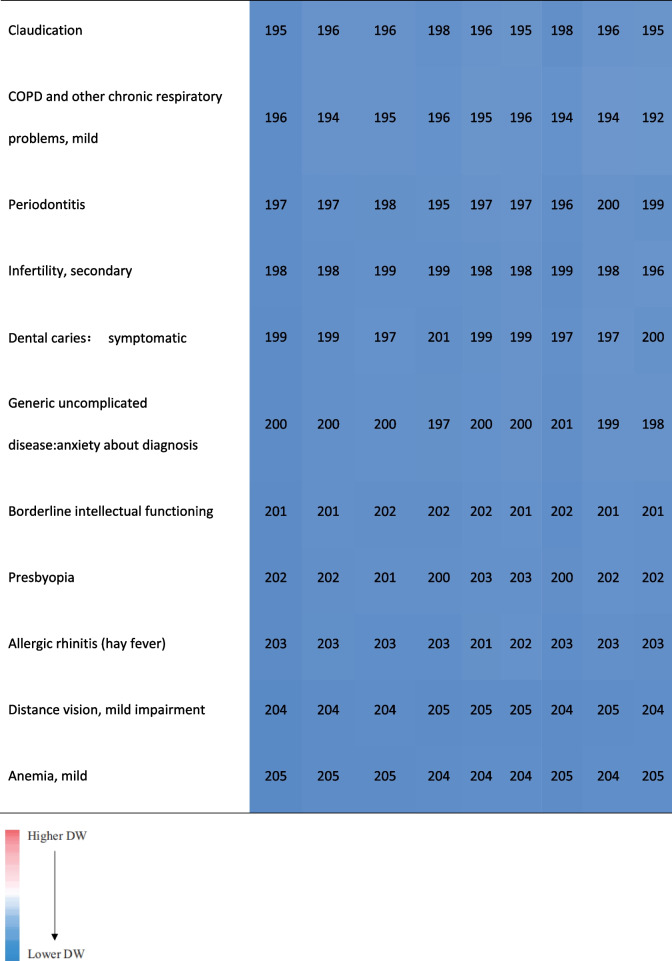
Comparison of the health states of the top 15 and the last 15 DWs in Anhui Province and their corresponding ranking of other provinces in China [17]

#### Comparison with Asian countries and GBD 2013

Supplementary Figure SF2 showed the DWs of Anhui province were highly correlated (*p* < 0.001) with that of China (r = 0.989), GBD2013 (r = 0.871) and Japan (r = 0.861). Supplementary Figure SF3, SF4 and SF5 showed the comparison results between Anhui and other studies respectively. More details were shown in Supplementary Table S3.

Compared with Chinese DWs, none of Anhui's DW was 2 times or more. The DW for 8 (3.88%) health states in China was 2 times or more than that in Anhui, of which there are 2 states with a difference of more than 3 times, mild impairment of distance vision and mild anemia respectively(SF3).

Compared with Japanese DWs, 32 (19.88%) states showed twice as much differences or more, half of which were higher than in Japan, and 4 of them in Anhui province were more than 3 times. 6 were only 1/3 or even 1/6 of Japanese DW (SF4).The largest difference was the DW of herpes zoster(0.181[0.123–0.257]) in Japan which is 6.70 times higher than that of Anhui.

Compared with GBD 2013, 37 (21.51%) states showed twice as much differences or more, of which 24 (64.86%) had a higher DW in Anhui province, and the DW of 8 states of Anhui province were more than 3 times, the largest difference was the DW of mild Cannabis dependence of Anhui province (0.405[0.357–0.459]), which is 10.38 times higher than the value of GBD 2013(0.039[0.024–0.060]). In addition, the DWs of Anhui province in the headache of tension type, mild amphetamine dependence, and symptomatic internal nematode infections were more than 4 times that of GBD2013. That for bulimia nervosa, mild episode of major depressive disorder, moderate motor plus cognitive impairments, and attention deficit hyperactivity disorder were only 1/3 of GBD2013 DW (SF5).

In terms of ranking order, Anhui's ranking results were very similar to China, but far from GBD 2013 and Japan. The difference between their DWs still visible to the naked eye. Among the health states of the top 15 and the last 15 DWs in Anhui province, about 43% or 57% of them appeared in GBD 2013 and Japan respectively.The DW ranked first in GBD2013 or Japan was different from Anhui province and China. The DWs ranked last 2 in theses studies were the same (Table 3). 46.67% of the top 15 DWs in Japan belonged to the category of mental, behavioral, and substance use disorders, which was similar to the results in Anhui and China. Most of the top 15 DWs in GBD 2013 belonged to the categories of infectious disease and cancer.

**Table 3.**
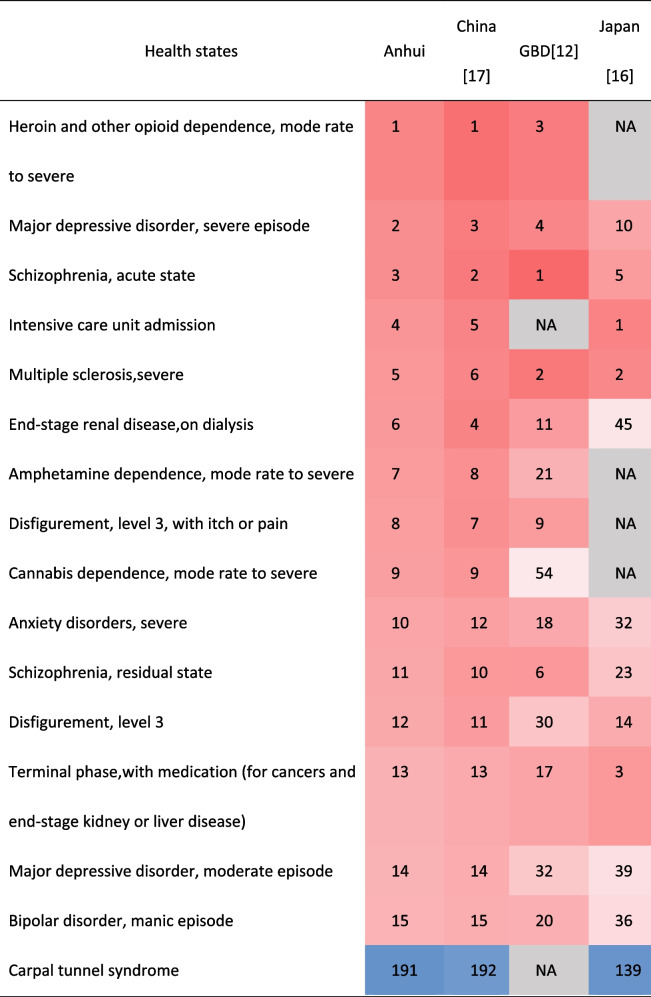

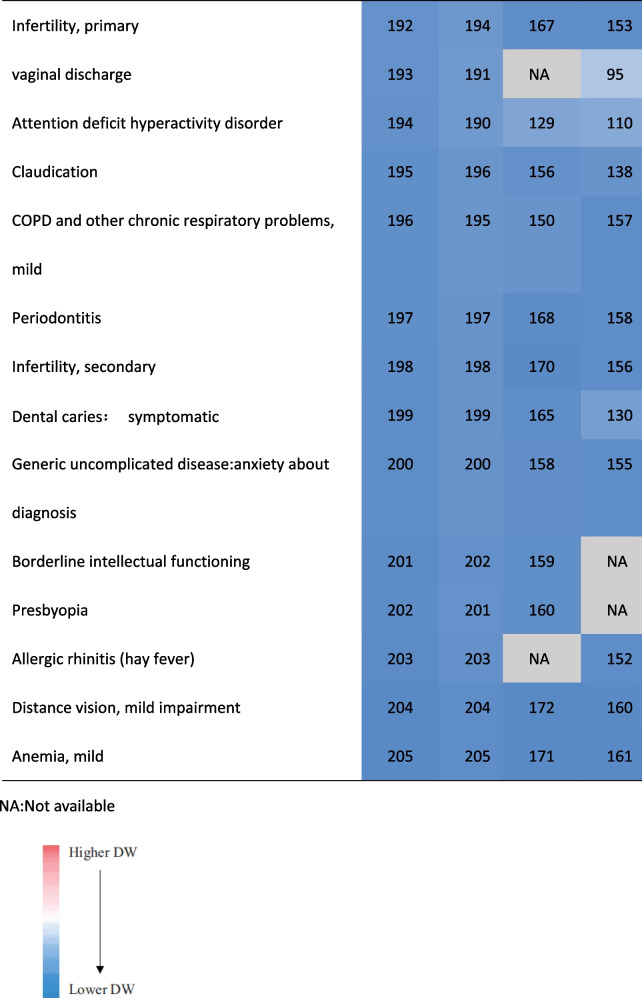
Comparison of the health states of the top 15 and the last 15 DW in Anhui province and their corresponding ranking in GBD2013 and Aisan countries

## Discussion

Based on the sample data obtained from the voluntary participants of the general population in Anhui Province, this study analyzed the DWs of the general population in Anhui province and compared it with the results of other provinces in China, the whole country, GBD and Japan. In addition to the same conclusions of other studies which the DWs were highly correlated with GBD, we also had new findings.

The variation range of DWs for health states in Anhui was very close to that in Japan [16]. According to the numbers of health states with large differences in DWs and the ranking of DWs, Anhui’s results were still less different from China and other provinces [17]. The DWs for mild impairment distance vision and mild anemia in other provinces except Henan were more than 3 times. Considering the very similar composition in gender, age and education level of participants and cultural backgrounds, is there really such a big difference? After further comparison with Anhui’s DWs in the Chinese study, surprisingly, the DWs of those 2 health states were still 3 times, although the Chinese study included only the results of 6,258 participants in Anhui province [17]. So can it be that different anchoring methods lead to different results? The Chinese study was anchored by the results of Chinese population health equivalence (PHE), not the results of GBD which both this study and the Japanese study adopted. We suspected that a higher DW was obtained by using PHE data to anchor. Consistent with the research results of four European countries and Japan, we also considered that the method of anchoring with PHE may not be applicable to the general population survey based on the network.Unfortunately, we cannot directly provide sufficient evidence to prove this, because we failed to obtain provincial PHE data.

Different from the results of domestic provinces and China, the health states numbers overlapped significantly less in the top 15 and the last 15 DWs in GBD and Japan and the ranking order was also quite different. The health state ranked first DW in Anhui, China and domestic provinces was severe heroin and other opioid dependence which ranked 3rd in GBD 2013 [12]. The DWs of health states were generally high if the name of the health state contained the word “dependence” in Anhui, China and domestic provinces. The DW of mild cannabis dependence, mild amphetamine dependence, mild cocaine dependence of Anhui province was 10.38 times, 4.41 times and 2.81 times higher than that of GBD 2013 [12] respectively. Different regions had different policies and cultural backgrounds, and people may have different perceptions for the same problem [12, 13, 16–22]. Take cannabis dependence, cannabis use was more common in high-income countries in recent years [23–25]. In Anhui, even in China, it was not widespread or even banned unless using for medical purposes. Take another example, both primary and secondary infertility in Anhui and China were 2 or 3 times than that in GBD2013. There is an old saying in China that there are three ways to be unfilial, the worst is not to produce off-spring. In the case of "hearing loss", the DWs were 0.019 for mild, 0.067 for moderate, 0.288 for severe, 0.245 for profound, and 0.166 for complete hearing loss. After reaching a certain degree, DW did not increased with the increase of severity, similar results were found only in Chinese study [17], this may be related to the concept, just as the meaning of "too much is not enough" in Chinese idiom.

WHO pointed out that the focus of the survey in developing countries was different from the developed countries [26]. The health states involved in each study were not completely unified, the number of injury related health states was limited in this study. This may also be the reason for the inconsistency of DW ranking and the first health state in each study. Other differences may be attributed to different ethnicity, health states at the time of the survey or self-perception [21, 22, 27].

Interestingly, most of the health states with top 15 DWs in Asian countries or regions belonged to the category of mental, behavioral, and substance use disorders which was significantly different from GBD 2013. Compared with Anhui’s DWs, the numbers of health states showed greater differences and accounted for about 10% in China and domestic provinces, 19.88% in Japan and 21.51% in GBD 2103 respectively. The differences of DWs in neighboring provinces were smaller than other geographically distant provinces or countries. Is the geographical distance also related to the difference of DW? This deserves further study.This finding further confirmed that the differences among similar-cultural regions were smaller than cross-cultural regions.

There was no gold standard for measurement methods. The visual analogue scale, time trade-off, person trade-off, standard gamble, interpolation, rank-based valuation techniques, PC and mapping function methods [1, 6, 9, 13, 15, 17, 28–30] could be used to measure health states. Each of them has different properties which may be affect the preferences that are measured [6]. The same health states in the same country had different values due to different methods [6, 13, 15].Considering that the gold standards were always absent in the measurement method,the contents of health states differed, the estimation criteria of comorbidity were loose, So, how can different valuation methods be equivalent? The main topic of the study on burden of disease is to obtain comprehensive, complete, consistent and comparable data. DW research was meant essentially to achieve this goal, but because of these problems, the final results may not be comparable. DW research methods have evolved over time, more and more DW research used PC techniques after 2011 [31]. Because it integrates the advantages of the distribution of the DWs of the health states, the pearson correlation coefficient, simplicity of the analysis, and basic generic health state layer descriptions, PC techniques has been proved to be utility, fairly straight-forward and simple method [2, 3, 13–16, 28]. However, generic descriptions should be used in conjunction with disease-specific descriptions to enhance standardized systematic descriptions of health states [31]. In addition, more evidence is needed to demonstrate the impact of anchoring methods on the results. Like the results of GBD2010 [2] and GBD2013 [12], this study found that PC responses were largely consistent across very distinct settings, its’ robustness was further verified, but the exceptions do need to be faced squarely. More DWs data in different settings need to be collected, pooled use for the global DW set in the GBD study in future to obtain comparable DWs or burden of disease estimates for all countries or all regions.

## Conclusions

There were differences in DWs in different countries or regions. The differences within the same countries and regions were smaller than those between different ones. And the differences of DWs in neighboring provinces were smaller than other geographically distant provinces or countries. Global or national DWs may not represent provincial levels, especially in the absence of gold standards. And there is an urgent need for relevant gold standards,which can at least eliminate the differences of the DWs caused by different measurement methods or research contents, even if there is no better way to solve the differences caused by cross-cultural backgrounds for the time being.

### Strengths and limitations

The first strength is that this study has enough samples at provincial level. Secondly, the present study obtained high-quality PC data. Thirdly, through the detailed comparison with other studies, we got some meaningful results, which will provide a new basis for the measurement of DWs in the future. However, this study still had limitations such as poor-quality PHE data and too little health states related to injury. This study cannot avoid a common problem of all online surveys. Most of the participants were higher educated groups, which may affect the provincial representation. And this study had no municipal representative data and comorbidity was not considered. These deficiencies need further research and improvement.

## Supplementary Information

**Additional file 1.** Supplementary Material.

## Data Availability

The data and materials that support the findings of this study are unavailable for public access because informed consent to share said data (beyond the research team) was not obtained from study participants, but are available from the corresponding author on reasonable request.

## Abbreviations

*DWs*: Disability weights
*GBD*: Global burden of disease
*DALYs*: Disability-adjusted life years
*PC*: Paired comparison
*UI*: Uncertainty interval
*PHE*: Population health equivalence
