# Supplementary material for: Can global or national disability weights represent provincial level?
Source: BMC Public Health. 2023 Mar 10;23:461. doi: 10.1186/s12889-022-14347-7 (PMC9999537; doi:10.1186/s12889-022-14347-7)
Supplement: Supplementary file 1 — Additional file 1. Supplementary Material. [file 12889_2022_14347_MOESM1_ESM.docx]

Supplementary Material

# **Supplementary Table S1 Lay** **descriptions for 206 health states in the study of Anhui Province in China**

| id | Health states | Health state lay description |
| --- | --- | --- |
| 1 | Infectious disease,Acute episode, mild | has a low fever and mild discomfort, but no difficulty with daily activities. |
| 2 | Infectious disease,Acute episode, moderate | has a fever and aches, and feels weak, which causes some difficulty with daily activities. |
| 3 | Infectious disease,Acute episode, severe | has a high fever and pain, and feels very weak, which causes great difficulty with daily activities. |
| 4 | Infectious disease,Post-acute effects (fatigue, emotional lability, and insomnia) | is always tired and easily upset. The person feels pain all over the body and is depressed. |
| 5 | Diarrhoea,Mild | has diarrhea three or more times a day with occasional discomfort in the belly. |
| 6 | Diarrhoea,Moderate | has diarrhea three or more times a day, with painful cramps in the belly and feeling thirsty |
| 7 | Diarrhoea,Severe | has diarrhea three or more times a day with severe belly cramps. The person is very thirsty and feels nauseous and tired. |
| 8 | Epididymo-orchitis | has swelling and tenderness in the testicles and pain during urination. |
| 9 | Herpes zoster | has a blistering skin rash that causes pain, with some burning and itching. |
| 10 | HIV: symptomatic, pre-AIDS | has weight loss, fatigue, and frequent infections. |
| 11 | HIV/AIDS: receiving antiretroviral treatment | has occasional fevers and infections. The person takes daily medication that sometimes causes diarrhea. |
| 12 | AIDS: not receiving antiretroviral treatment | has severe weight loss, weakness, fatigue, cough and fever, and frequent infections, skin rashes and diarrhea. |
| 13 | Intestinal nematckle infections: symptomatic | has cramping pain and a bloated feeling in the belly. |
| 14 | Lymphatic filariasis: symptomatic | has swollen legs with hard and thick skin, which causes difficulty in moving around. |
| 15 | Ear pain | has an ear ache that causes some difficulty with daily activities. |
| 16 | Tuberculosis,Without HIV infection | has a persistent cough and fever, is short of breath, feels weak, and has lost a lot of weight. |
| 17 | Tuberculosis,With HIV infection | has a persistent cough and fever, shortness of breath, night sweats, weakness and fatigue and severe weight loss. |
| 18 | Cancer,Diagnosis and primary treatment | has pain, nausea, fatigue, weight loss and high anxiety. |
| 19 | Cancer,Metastatic | has severe pain, extreme fatigue, weight loss and high anxiety. |
| 20 | Terminal phase,With medication (for cancers and end-stage kidney or liver disease) | has lost a lot of weight and regularly uses strong medication to avoid constant pain. The person has no appetite, feels nauseous, and needs to spend most of the day in bed. |
| 21 | Terminal phase,Without medication (for cancers and end-stage kidney or liver disease) | has lost a lot of weight and has constant pain. The person has no appetite, feels nauseous, and needs to spend most of the day in bed. |
| 22 | Cardiovascular and circulatory disease,Acute myocardial infarction,Days 1-2 | has severe chest pain that becomes worse with any physical activity, feels nauseous, short of breath, and very anxious. |
| 23 | Cardiovascular and circulatory disease,Acute myocardial infarction,Days 3-28 | gets short of breath after heavy physical activity, and tires easily, but has no problems when at rest. The person has to take medication every day and has some anxiety. |
| 24 | Angina pectoris,Mild | has chest pain that occurs with strenuous physical activity, such as running or lifting heavy objects. After a brief rest, the pain goes away. |
| 25 | Angina pectoris,Moderate | has chest pain that occurs with moderate physical activity, such as walking uphill or more than half a kilometer (around a quarter mile) on level ground. After a brief rest, the pain goes away. |
| 26 | Angina pectoris,Severe | has chest pain that occurs with minimal physical activity, such as walking only a short distance. After a brief rest, the pain goes away. The person avoids most physical activities because of the pain. |
| 27 | Cardiac conduction disorders and cardiac dysrhythmias | has periods of rapid and irregular heartbeats and occasional fainting. |
| 28 | Claudication | has cramping pains in the legs after walking a medium distance. The pain goes away after a short rest. |
| 29 | Heart failure,Mild | is short of breath and easily tires with moderate physical activity, such as walking uphill or more than a quarter mile on level ground. The person feels comfortable at rest or during activities requiring less effort. |
| 30 | Heart failure,Moderate | is short of breath and easily tires with minimal physical activity, such as walking only a short distance. The person feels comfortable at rest but avoids moderate activity. |
| 31 | Heart failure,Severe | is short of breath and feels tired when at rest. The person avoids any physical activity, for fear of worsening the breathing problems. |
| 32 | Stroke,Long-term consequences, mild | has some difficulty in moving around and some weakness in one hand, but is able to walk without help. |
| 33 | Stroke,Long-term consequences, moderate | has some difficulty in moving around, and in using the hands for lifting and holding things, dressing and grooming. |
| 34 | Stroke,Long-term consequences, moderate, plus cognition problems | has some difficulty in moving around, in using the hands for lifting and holding things, dressing and grooming, and in speaking. The person is often forgetful and confused. |
| 35 | Stroke,Long-term consequences severe | is confined to bed or a wheelchair, has difficulty speaking and depends on others for feeding, toileting and dressing. |
| 36 | Stroke,Long-term consequences, severe, plus cognition problems | is confined to bed or a wheelchair, depends on others for feeding, toileting and dressing, and has difficulty speaking, thinking clearly and remembering things. |
| 37 | Diabetes and digestive and genitourinary disease,Diabetic neuropathy | has pain, tingling and numbness in the arms, legs, hands and feet. The person sometimes gets cramps and muscle weakness. |
| 38 | Diabetes and digestive and genitourinary disease,Chronic kidney disease (stage 4) | tires easily, has nausea, reduced appetite and difficulty sleeping. |
| 39 | With kidney transplantation | sometimes feels tired and down, and has some difficulty with daily activities. |
| 40 | On dialysis | is tired and has itching, cramps, headache, joint pains and shortness of breath. The person needs intensive medical care every other day lasting about half a day. |
| 41 | Decompensated liver cirrhosis | has a swollen belly and swollen legs. The person feels weakness, fatigue and loss of appetite. |
| 42 | Gastric bleeding | vomits blood and feels nauseous. |
| 43 | Crohn's disease or ulcerative colitis | has cramping abdominal pain, has diarrhea several times a day, and feels very tired for two months every year. When the person does not have symptoms, there is anxiety about them returning. |
| 44 | Benign prostatic hypertrophy: symptomatic | feels the urge to urinate frequently, but when passing urine it comes out slowly and sometimes is painful. |
| 45 | Urinary incontinence | cannot control urinating. |
| 46 | Stress incontinence | This person loses small amounts of urine without meaning to when coughing, sneezing, laughing or during physical exercise |
| 47 | Impotence | has difficulty in obtaining or maintaining an erection. |
| 48 | Infertility, Primary | wants to have a child and has a fertile partner, but the couple cannot conceive. |
| 49 | Infertility, Secondary | has at least one child, and wants to have more children. The person has a fertile partner, but the couple cannot conceive. |
| 50 | Asthma, Controlled | has wheezing and cough once a month, which does not cause difficulty with daily activities. |
| 51 | Asthma, Partly controlled | has wheezing and cough once a week, which causes some difficulty with daily activities. |
| 52 | Asthma, Uncontrolled | has wheezing, cough and shortness of breath more than twice a week, which causes difficulty with daily activities and sometimes wakes the person at night. |
| 53 | COPD and other chronic respiratory problems, Mild | has cough and shortness of breath after heavy physical activity, but is able to walk long distances and climb stairs. |
| 54 | COPD and other chronic respiratory problems, Moderate | has cough, wheezing and shortness of breath, even after light physical activity. The person feels tired and can walk only short distances or climb only a few stairs. |
| 55 | COPD and other chronic respiratory problems, Severe | has cough, wheezing and shortness of breath all the time. The person has great difficulty walking even short distances or climbing any stairs, feels tired when at rest, and is anxious. |
| 56 | Dementia, Mild | has some trouble remembering recent events, and finds it hard to concentrate and make decisions and plans |
| 57 | Dementia, Moderate | has memory problems and confusion, feels disoriented, at times hears voices that are not real, and needs help with some daily activities. |
| 58 | Dementia, Severe | has complete memory loss; no longer recognizes close family members; and requires help with all daily activities. |
| 59 | Migraine | has severe, throbbing head pain and nausea that cause great difficulty in daily activities and sometimes confine the person to bed. Moving around, light, and noise make it worse. |
| 60 | Tension-type | has a moderate headache that also affects the neck, which causes difficulty in daily activities. |
| 61 | Medication overuse | has daily headaches, felt as dull pain and often lasting all day, with poor sleep, nausea and fatigue. The person takes medicine for the headaches, which provides little relief but is needed to avoid having worse symptoms. |
| 62 | Multiple sclerosis,Mild | has mild loss of feeling in one hand, is a little unsteady while walking, has slight loss of vision in one eye, and often needs to urinate urgently. |
| 63 | Multiple sclerosis,Moderate | needs help walking, has difficulty with writing and arm coordination, has loss of vision in one eye and cannot control urinating. |
| 64 | Multiple sclerosis,Severe | has slurred speech and difficulty swallowing. The person has weak arms and hands, very limited and stiff leg movement, has loss of vision in both eyes and cannot control urinating. |
| 65 | Epilepsy, Severe (seizures once per month or more) | has sudden seizures one or more times each month, with violent muscle contractions and stiffness, loss of consciousness, and loss of urine or bowel control. Between seizures the person has memory loss and difficulty concentrating. |
| 66 | Epilepsy, less severe (seizures less than once per month) | has sudden seizures two to five times a year, with violent muscle contractions and stiffness, loss of consciousness, and loss of urine or bowel control. |
| 67 | Parkinson disease, Mild | has mild tremors and moves a little slowly, but is able to walk and do daily activities without assistance. |
| 68 | Parkinson disease, Moderate | has moderate tremors and moves slowly, which causes some difficulty in walking and daily activities. The person has some trouble swallowing, talking, sleeping, and remembering things. |
| 69 | Parkinson disease, Severe | has severe tremors and moves very slowly, which causes great difficulty in walking and daily activities. The person falls easily and has a lot of difficulty talking, swallowing, sleeping, and remembering things. |
| 70 | Alcohol use disorder, Very mild | drinks alcohol daily and has difficulty controlling the urge to drink. When sober, the person functions normally. |
| 71 | Alcohol use disorder, Mild | drinks a lot of alcohol and sometimes has difficulty controlling the urge to drink. While intoxicated, the person has difficulty performing daily activities. |
| 72 | Alcohol use disorder, Moderate | drinks a lot, gets drunk almost every week and has great difficulty controlling the urge to drink. Drinking and recovering cause great difficulty in daily activities, sleep loss, and fatigue. |
| 73 | Alcohol use disorder, Severe | gets drunk almost every day and is unable to control the urge to drink. Drinking and recovering replace most daily activities. The person has difficulty thinking, remembering and communicating, and feels constant pain and fatigue. |
| 74 | Fetal alcohol syndrome,Mild | is a little slow in developing physically and mentally, which causes some difficulty in learning but no other difficulties in daily activities. |
| 75 | Fetal alcohol syndrome,Moderate | is slow in developing physically and mentally, which causes some difficulty in daily activities. |
| 76 | Fetal alcohol syndrome,Severe | is very slow in developing physically and mentally, which causes great difficulty in daily activities. |
| 77 | Cannabis dependence, Mild | uses marijuana at least once a week and has some difficulty controlling the habit. When not using, the person functions normally. |
| 78 | Cannabis dependence, Mode rate to severe | uses marijuana daily and has difficulty controlling the habit. The person sometimes has mood swings, anxiety and hallucinations, and has some difficulty in daily activities. |
| 79 | Amphetamine dependence, Mild | uses stimulants (drugs) at least once a week and has some difficulty controlling the habit. When not using, the person functions normally. |
| 80 | Amphetamine dependence, Mode rate to severe | uses stimulants (drugs) and has difficulty controlling the habit. sometimes has depression, hallucinations and mood swings, and has difficulty in daily activities. |
| 81 | Cocaine dependence, Mild | uses cocaine at least once a week and has some difficulty controlling the habit. When not using, the person functions normally. |
| 82 | Cocaine dependence, Mode rate to severe | uses cocaine and has difficulty controlling the habit. The person sometimes has mood swings, anxiety, paranoia, hallucinations and sleep problems, and has some difficulty in daily activities. |
| 83 | Heroin and other opioid dependence, Mild | uses heroin (or methadone) every day and has difficulty controlling the habit. When not using, the person functions normally. |
| 84 | Heroin and other opioid dependence, Mode rate to severe | uses heroin daily and has difficulty controlling the habit. When the effects wear off, the person feels severe nausea, agitation, vomiting and fever. The person has a lot of difficulty in daily activities. |
| 85 | Anxiety disorders, Mild | feels mildly anxious and worried, which makes it slightly difficult to concentrate, remember things, and sleep. The person tires easily but is able to perform daily activities. |
| 86 | Anxiety disorders, Moderate | feels anxious and worried, which makes it difficult to concentrate, remember things, and sleep. The person tires easily and finds it difficult to perform daily activities. |
| 87 | Anxiety disorders, Severe | constantly feels very anxious and worried, which makes it difficult to concentrate, remember things and sleep. The person has lost pleasure in life and thinks about suicide. |
| 88 | Major depressive disorder, Mild episode | feels persistent sadness and has lost interest in usual activities. The person sometimes sleeps badly, feels tired, or has trouble concentrating but still manages to function in daily life with extra effort. |
| 89 | Major depressive disorder, Moderate episode | has constant sadness and has lost interest in usual activities. The person has some difficulty in daily life, sleeps badly, has trouble concentrating, and sometimes thinks about harming himself (or herself). |
| 90 | Major depressive disorder, Severe episode | has overwhelming, constant sadness and cannot function in daily life. The person sometimes loses touch with reality and wants to harm or kill himself (or herself). |
| 91 | Bipolar disorder, Manic episode | is hyperactive, hears and believes things that are not real, and engages in impulsive and aggressive behavior that endanger the person and others. |
| 92 | Bipolar disorder, Residual state | has mild mood swings, irritability and some difficulty with daily activities. |
| 93 | Schizophrenia, Acute state | hears and sees things that are not real and is afraid, confused, and sometimes violent. The person has great difficulty with communication and daily activities, and sometimes wants to harm or kill himself (or herself). |
| 94 | Schizophrenia, Residual state | hears and sees things that are not real and has trouble communicating. The person can be forgetful, has difficulty with daily activities, and thinks about hurting himself (or herself). |
| 95 | Anorexia nervosa | feels an overwhelming need to starve and exercises excessively to lose weight. The person is very thin, weak and anxious. |
| 96 | Bulimia nervosa | has uncontrolled overeating followed by guilt, starving, and vomiting to lose weight |
| 97 | Attention deficit hyperactivity disorder | is hyperactive and has difficulty concentrating, remembering things, and completing tasks. |
| 98 | Conduct disorder | has frequent behavior problems, which are sometimes violent. The person often has difficulty interacting with other people and feels irritable. |
| 99 | Borderline intellectual functioning | is slow in learning at school. As an adult, the person has some difficulty doing complex or unfamiliar tasks but otherwise functions independently. |
| 100 | Intellectual disability/mental retardation, Mild | has low intelligence and is slow in learning at school. As an adult, the person can live independently, but often needs help to raise children and can only work at simple supervised jobs. |
| 101 | Intellectual disability/mental retardation, Moderate | has low intelligence, and is slow in learning to speak and to do even simple tasks. As an adult, the person requires a lot of support to live independently and raise children. The person can only work at the simplest supervised jobs. |
| 102 | Intellectual disability/mental retardation, Severe | has very low intelligence and cannot speak more than a few words, needs constant supervision and help with most daily activities, and can do only the simplest tasks. |
| 103 | Intellectual disability/mental retardation, Profound | has very low intelligence, has almost no language, and does not understand even the most basic requests or instructions. The person requires constant supervision and help for all activities. |
| 104 | Hearing loss, Mild | has great difficulty hearing and understanding another person talking in a noisy place (for example, on an urban street). |
| 105 | Hearing loss, Moderate | is unable to hear and understand another person talking in a noisy place (for example, on an urban street), and has difficulty hearing another person talking even in a quiet place or on the phone. |
| 106 | Hearing loss, Severe | is unable to hear and understand another person talking, even in a quiet place, and unable to take part in a phone conversation. Difficulties with communicating and relating to others cause emotional impact at times (for example worry or depression). |
| 107 | Hearing loss, Profound | is unable to hear and understand another person talking, even in a quiet place, is unable to take part in a phone conversation, and has great difficulty hearing anything in any other situation. Difficulties with communicating and relating to others often cause worry, depression, and loneliness. |
| 108 | Hearing loss, Complete | cannot hear at all in any situation, including even the loudest sounds, and cannot communicate verbally or use a phone. Difficulties with communicating and relating to others often cause worry, depression or loneliness. |
| 109 | Hearing loss, Mild, with ringing | has great difficulty hearing and understanding another person talking in a noisy place (for example, on an urban street), and sometimes has annoying ringing in the ears. |
| 110 | Hearing loss, Moderate, with ringing | is unable to hear and understand another person talking in a noisy place (for example, on an urban street), and has difficulty hearing another person talking even in a quiet place or on the phone, and has annoying ringing in the ears for more than 5 minutes at a time, almost everyday. |
| 111 | Hearing loss, Severe, with ringing | is unable to hear and understand another person talking, even in a quiet place, and unable to take part in a phone conversation, and has annoying ringing in the ears for more than 5 minutes at a time, almost everyday. Difficulties with communicating and relating to others cause emotional impact at times (for example worry or depression). |
| 112 | Hearing loss, Profound, with ringing | is unable to hear and understand another person talking, even in a quiet place, is unable to take part in a phone conversation, has great difficulty hearing anything in any other situation, and has annoying ringing in the ears for more than 5 minutes at a time, several times a day. Difficulties with communicating and relating to others often cause worry, |
| 113 | Hearing loss, Complete, with ringing | cannot hear at all in any situation, including even the loudest sounds, and cannot communicate verbally or use a phone, and has very annoying ringing in the ears for more than half of the day. Difficulties with communicating and relating to others often cause worry, depression or loneliness. |
| 114 | Distance vision, Mild impairment | has some difficulty with distance vision, for example reading signs, but no other problems with eyesight. |
| 115 | Distance vision, Moderate impairment | has vision problems that make it difficult to recognize faces or objects across a room. |
| 116 | Distance vision, Severe impairment | has severe vision loss, which causes difficulty in daily activities, some emotional impact (for example worry), and some difficulty going outside the home without assistance. |
| 117 | Distance vision, Blindness | is completely blind, which causes great difficulty in some daily activities, worry and anxiety, and great difficulty going outside the home without assistance. |
| 118 | Distance vision, Monocular impairment | is blind in one eye and has difficulty judging distances |
| 119 | Presbyopia | has difficulty seeing things that are nearer than 3 feet, but has no difficulty with seeing things at a distance. |
| 120 | Low back pain, Mild | has mild back pain, which causes some difficulty dressing, standing, and lifting things. |
| 121 | Low back pain,Moderate | has moderate back pain, which causes difficulty dressing, sitting, standing, walking, and lifting things. |
| 122 | Low back pain,Severe, without leg pain | has severe back pain, which causes difficulty dressing, sitting, standing, walking, and lifting things. The person sleeps poorly and feels worried. |
| 123 | Low back pain,Severe, with leg pain | has severe back and leg pain, which causes difficulty dressing, sitting, standing, walking, and lifting things. The person sleeps poorly and feels worried. |
| 124 | Low back pain,Most severe, without leg pain | has constant back pain, which causes difficulty dressing, sitting, standing, walking, and lifting things. The person sleeps poorly, is worried, and has lost some enjoyment in life. |
| 125 | Low back pain,Most severe, with leg pain | has constant back and leg pain, which causes difficulty dressing, sitting, standing, walking, and lifting things. The person sleeps poorly, is worried, and has lost some enjoyment in life. |
| 126 | Neck pain, Mild | has neck pain, and has difficulty turning the head and lifting things. |
| 127 | Neck pain,Moderate | has constant neck pain, and has difficulty turning the head, holding arms up, and lifting things |
| 128 | Neck pain, Severe | has severe neck pain, and difficulty turning the head and lifting things. The person gets headaches and arm pain, sleeps poorly, and feels tired and worried. |
| 129 | Neck pain,Most severe | has constant neck pain and arm pain, and difficulty turning the head, holding arms up, and lifting things. The person gets headaches, sleeps poorly, and feels tired and worried. |
| 130 | Musculoskeletal problems, Legs, mild | has pain in the leg, which causes some difficulty running, walking long distances, and getting up and down. |
| 131 | Musculoskeletal problems, Legs, moderate | has moderate pain in the leg, which makes the person limp, and causes some difficulty walking, standing, lifting and carrying heavy things, getting up and down and sleeping. |
| 132 | Musculoskeletal problems, Legs, severe | has severe pain in the leg, which makes the person limp and causes a lot of difficulty walking, standing, lifting and carrying heavy things, getting up and down, and sleeping. |
| 133 | Musculoskeletal problems, Arms, mild | has mild pain and stiffness in the arms and hands. The person has some difficulty lifting, carrying and holding things. |
| 134 | Musculoskeletal problems, Arms, moderate | has moderate pain and stiffness in the arms and hands, which causes difficulty lifting, carrying, and holding things, and trouble sleeping because of the pain. |
| 135 | Musculoskeletal problems, Generalized, moderate | has pain and deformity in most joints, causing difficulty moving around, getting up and down, and using the hands for lifting and carrying. The person often feels fatigue. |
| 136 | Musculoskeletal problems, Generalized, severe | has severe, constant pain and deformity in most joints, causing difficulty moving around, getting up and down, eating, dressing, lifting, carrying and using the hands. The person often feels sadness, anxiety and extreme fatigue. |
| 137 | Gout: acute | has severe pain and swelling in the leg, making it very difficult to get up and down, stand, walk, lift, and carry heavy things. The person has trouble sleeping because of the pain. |
| 138 | Amputation, One arm:long term, without treatment | has lost one hand and part of the arm, leaving pain and tingling in the stump. The person needs help from others to lift objects or do daily activities such as cooking. |
| 139 | Concussion | This person has episodes of headaches, dizziness, nausea and difficulty concentration. |
| 140 | Spinal cord lesion, Below neck： treated | is paralyzed from the waist down, cannot feel or move the legs and has difficulties with urine and bowel control. The person uses a wheelchair to move around. |
| 141 | Abdominopelvic problem, Mild | has some pain in the belly that causes nausea but does not interfere with daily activities. |
| 142 | Abdominopelvic problem, Moderate | has pain in the belly and feels nauseous. The person has difficulties with daily activities. |
| 143 | Abdominopelvic problem, Severe | has severe pain in the belly and feels nauseous. The person is anxious and unable to carry out daily activities. |
| 144 | Anemia, Mild | feels slightly tired and weak at times, but this does not interfere with normal daily activities. |
| 145 | Anemia, Moderate | feels moderate fatigue, weakness, and shortness of breath after exercise, making daily activities more difficult. |
| 146 | Anemia, Severe | feels very weak, tired and short of breath, and has problems with activities that require physical effort or deep concentration. |
| 147 | Periodontitis | has minor bleeding of the gums from time to time, with mild discomfort. |
| 148 | Dental caries: symptomatic | has a toothache, which causes some difficulty in eating. |
| 149 | Severe tooth loss | has lost more than 20 teeth including front and back, and has great difficulty in eating meat, fruits, and vegetables. |
| 150 | Disfigurement, Level 1 | has a slight, visible physical deformity that others notice, which causes some worry and discomfort. |
| 151 | Disfigurement, Level 2 | has a visible physical deformity that causes others to stare and comment. As a result, the person is worried and has trouble sleeping and concentrating. |
| 152 | Disfigurement, Level 3 | has an obvious physical deformity that makes others uncomfortable, which causes the person to avoid social contact, feel worried, sleep poorly, and think about suicide. |
| 153 | Disfigurement, Level 1, with itch or pain | has a slight, visible physical deformity that is sometimes sore or itchy. Others notice the deformity, which causes some worry and discomfort. |
| 154 | Disfigurement, Level 2, with itch or pain | has a visible physical deformity that is sore and itchy. Other people stare and comment, which causes the person to worry. The person has trouble sleeping and concentrating. |
| 155 | Disfigurement, Level 3, with itch or pain | has an obvious physical deformity that is very painful and itchy. The physical deformity makes others uncomfortable, which causes the person to avoid social contact, feel worried, sleep poorly, and think about suicide. |
| 156 | Generic uncomplicated disease:Worry and daily medication | has a chronic disease that requires medication every day and causes some worry but minimal interference with daily activities. |
| 157 | Generic uncomplicated disease:Anxiety about diagnosis | has a disease diagnosis that causes some worry but minimal interference with daily activities. |
| 158 | Kwashiorkor | is very tired and irritable and has diarrhea. |
| 159 | Severe wasting | is extremely skinny and has no energy. |
| 160 | Speech problems | has difficulty speaking, and others find it difficult to understand. |
| 161 | Motor impairment, Mild | has some difficulty in moving around but is able to walk without help. |
| 162 | Motor impairment, Moderate | has some difficulty in moving around, and difficulty in lifting and holding objects, dressing and sitting upright, but is able to walk without help. |
| 163 | Motor impairment, Severe | is unable to move around without help, and is not able to lift or hold objects, get dressed or sit upright. |
| 164 | Motor plus cognitive impairments,Mild | has some difficulty in moving around but is able to walk without help. The person is slow in learning at school. As an adult, the person has some difficulty doing complex or unfamiliar tasks but otherwise functions independently. |
| 165 | Motor plus cognitive impairments,Moderate | has some difficulty in moving around, holding objects, dressing and sitting upright, but can walk without help. The person has low intelligence and is slow in learning to speak and to do simple tasks. |
| 166 | Motor plus cognitive impairments,Severe | cannot move around without help, and cannot lift or hold objects, get dressed or sit upright. The person also has very low intelligence, speaks few words, and needs constant supervision and help with all daily activities. |
| 167 | Rectovaginal fistula | has an abnormal opening between her vagina and rectum causing flatulence and feces to escape through the vagina. The person gets infections in her vagina, and has pain when urinating. |
| 168 | Vesicovaginal fistula | has an abnormal opening between the bladder and the vagina, which makes her unable to control urinating. The woman is anxious and depressed. |
| 169 | Thrombocytopenic purpura | easily bruises and sometimes bleeds from the gums and nose; feels weak and has some difficulty with daily activities. |
| 170 | Hypothyroidism | has low energy and feels cold. |
| 171 | Hyperthyroidism | feels nervous, has palpitations, sweats a lot and has difficulty sleeping. |
| 172 | Vertigo | this person has short spells of dizziness and losing balance; when these spells are not there the person is worried it may happen again |
| 173 | Hearing loss, severe(Modified)^*^ | is unable to hear and understand another person talking, even in a quiet place, and unable to take part in a phone conversation. Difficulties with communicating and relating to others cause emotional impact at times (for example worry or depression). |
| 174 | Hearing loss, moderate(Modified)^*^ | is unable to hear and understand another person talking in a noisy place (for example, on an urban street), and has difficulty hearing another person talking even in a quiet place or on the phone. |
| 175 | Heart burn & reflux “GERD” | Often has a burning sensation in the back of the chest after eating. |
| 176 | Stress incontinence | loses small amounts of urine without meaning to when coughing, sneezing, laughing or during physical exercise. |
| 177 | Neck pain, moderate | has moderately severe neck pain, and difficulty turning the head and lifting things. The person gets headaches. |
| 178 | Allergic rhinitis (hay fever) | This person has an itchy running nose and frequently sneezes |
| 179 | Often has a burning sensation in the back of the chest after eating | A couple of times a day, this person has a burning sensation in the back of the chest as contents from the stomach moves back into the oesophagus |
| 180 | Constipation | this person passes stools infrequently and when it does happen it is painful |
| 181 | Haemorrhoids | this person loses some blood when passing stools and at times has pain around the anus |
| 182 | somatoform disorder | this person suffers from multiple symptoms of pain, stomach complaints or nerve problems for which no explanation can be found. The symptoms are real to the person and a source of worry |
| 183 | insomnia | this person has difficulty falling or staying asleep |
| 184 | sleep apnoea | this person while sleeping has short episodes of abnormally low breathing; the person does not notice this but may feel tired or sleepy during the day |
| 185 | Hypothyroidism | this person feels tired, has gained weight and feels cold |
| 186 | borderline personality disorder | this person has unstable moods, often does things without thinking about the consequences and has a tendency to view others and self as either very good or very bad. This leads to chaotic interpersonal relationships and causes the person to feel angry, anxious or depressed |
| 187 | harmful alcohol use | this person regularly gets drunk putting the person at risk of injuries and other harm to health |
| 188 | Hyperthyroidism | this person is feels nervous, has palpitations, sweats a lot and has difficulty sleeping |
| 189 | vaginal discharge | this woman has a vaginal discharge that sometimes causes itch |
| 190 | dyspareunia | this woman experiences pain during sexual intercourse |
| 191 | trigeminal neuralgia | this person has episodes of severe pain in the face |
| 192 | anal fissure/abcess/fistula | this person has pain around the anus that is worse when passing stools and sitting |
| 193 | Varicose veins | This person has swollen veins in the legs that sometimes cause some swelling of the feet |
| 194 | Carpal tunnel syndrome | This person feels pain, numbness or weakness in part of the hand during some activities such as knitting, driving or typing |
| 195 | Osteomyelitis | This person has severe pain in one leg causing difficulty in moving about |
| 196 | Shoulder lesions | This person has a painful shoulder that causes difficulty in using the arm |
| 197 | Irritable bowel syndrome | This person experiences abdominal pain, bloating, stomach rumbling, flatulence and an irregular bowel pattern. |
| 198 | Thrombocytopenic purpura196 | This person has bruising on the extremities and bleeding of the gums and nose and feels very weak. |
| 199 | Lymphogranuloma Venereum - local infection | This person has a painful lump in the groin. |
| 200 | Intensive care unit admission | This person is very ill, confined to bed; often unconscious or asleep, unable to speak and completely dependent on others. |
| 201 | Invasive device/drain | This person carries a drain, which may irritate locally and also hinders the patient when moving around. |
| 202 | Encephalopathy - moderate | has difficulty concentrating, confusion, fatigue and irritability. The person has difficulty with walking, speaking and personal care. |
| 203 | Encephalopathy - severe | has difficulty concentrating or speaking, confusion, fatigue and irritability. The person needs a great deal of help from others to do even basic daily activities such as eating and using the toilet, and the person is very limited in other activities. |
| 204 | Subacute sclerosing panencephalitis - phase 1 | has difficulty concentrating, some memory problems, confusion and irritability. |
| 205 | Subacute sclerosing panencephalitis - phase 2 | has difficulty concentrating, some memory problems, confusion and irritability. The person has difficulty controlling body movements and moving around. |
| 206 | Subacute sclerosing panencephalitis - phase 3 | is often asleep or unconscious; when awake cannot think or see clearly. The person needs a great deal of help from others to do even basic daily activities such as eating and using the toilet, and the person is very limited in other activities. |

**Supplementary Table S2 Basic characteristics of participants in Anhui province compared to the provincial population**

|  | Total population(%) | Web-based surveys(%, n=39446) | *P-value^d^* |
| --- | --- | --- | --- |
| Sex |  |  | 0.209 |
| Male | 50.41 ^a^ | 40.56 |  |
| Female | 49.59 ^a^ | 59.44 |  |
| Age(years) |  |  | 0.016 |
| 18-29 | 18.63 ^a^ | 21.64 |  |
| 30-49 | 30.68 ^a^ | 61.95 |  |
| 50-69 | 23.99 ^a^ | 16.40 |  |
| Region |  |  | 0.937 |
| Northern Anhui | 38.35 ^a^ | 36.28 |  |
| Central Anhui | 44.93 ^a^ | 45.43 |  |
| Southern Anhui | 16.72 ^a^ | 18.29 |  |
| Nation |  |  | 1.000 |
| Han nationality | 99.29^b^ | 98.87 |  |
| Ethnic minorities | 0.71^b^ | 1.13 |  |
| Educational level |  |  | <0.001* |
| Low | 73.16^c^ | 6.37 |  |
| Medium | 14.64 ^c^ | 26.22 |  |
| High | 12.20 ^c^ | 67.41 |  |

a: According to China disease prevention and control information system.

b: According to AnHui Statistical Yearbook-2020 (2019 population)

c: According to China Statistical Yearbook-2020 (Sample population of Anhui Province,2019)

# d: P values resulting from the Chi-square test or Fisher’s exact test

# Supplementary Table S3 **Comparison of Disability weights and 95% uncertainty intervals among Anhui, China^17^, GBD2013^12^ and Japan^16^**

| id | Health states | Anhui | China | GBD2013 | Japan |
| --- | --- | --- | --- | --- | --- |
| Infectious disease | | | | | |
|  | Infectious disease |  |  |  |  |
| 1 | Acute episode, mild | 0.018(0.015-0.023) | 0.027(0.002-0.113) | 0.006(0.002-0.012) | 0.012(0.005-0.022) |
| 2 | Acute episode, moderate | 0.150(0.126-0.182) | 0.137(0.054-0.273) | 0.051(0.032-0.074) | 0.424(0.289-0.577) |
| 3 | Acute episode, severe | 0.169(0.139-0.201) | 0.152(0.066-0.288) | 0.133(0.088-0.190) | 0.242(0.163-0.340) |
| 4 | Post-acute effects (fatigue, emotional lability, and insomnia) | 0.084(0.068-0.104) | 0.080(0.02-0.21) | 0.219(0.148-0.308) | 0.074(0.047-0.106) |
|  | Diarrhoea |  |  |  |  |
| 5 | Mild | 0.070(0.057-0.087) | 0.072(0.016-0.198) | 0.074(0.049-0.104) | 0.119(0.079-0.165) |
| 6 | Moderate | 0.175(0.146-0.209) | 0.151(0.064-0.286) | 0.188(0.125-0.264) | 0.250(0.170-0.345) |
| 7 | Severe | 0.301(0.259-0.346) | 0.259(0.17-0.368) | 0.247(0.164-0.348) | 0.387(0.263-0.517) |
| 8 | Epididymo-orchitis | 0.124(0.103-0.149) | 0.122(0.044-0.259) | 0.128(0.086-0.180) | 0.204(0.139-0.283) |
| 9 | Herpes zoster | 0.027(0.022-0.035) | 0.045(0.006-0.154) | 0.058(0.035-0.090) | 0.181(0.123-0.257) |
| 10 | HIV: symptomatic, pre-Al DS | 0.286(0.247-0.332) | 0.252(0.163-0.363) | 0.274(0.184-0.377) | 0.200(0.140-0.275) |
| 11 | HIV/AIDS: receiving antiretroviral treatment | 0.066(0.054-0.083) | 0.074(0.017-0.201) | 0.078(0.052-0.111) | 0.155(0.108-0.219) |
| 12 | AIDS: not receiving antiretroviral treatment | 0.392(0.345-0.443) | 0.367(0.309-0.432) | 0.582(0.406-0.743) | 0.394(0.268-0.527) |
| 13 | Intestinal nematckle infections: symptomatic | 0.110(0.089-0.133) | 0.112(0.037-0.248) | 0.027(0.015-0.043) | NA |
| 14 | Lymphatic filariasis: symptomatic | 0.162(0.135-0.194) | 0.153(0.067-0.289) | 0.109(0.073-0.154) | NA |
| 15 | Ear pain | 0.047(0.038-0.059) | 0.052(0.009-0.166) | 0.013(0.007-0.024) | 0.058(0.037-0.085) |
|  | Tuberculosis |  |  |  |  |
| 16 | Without HIV infection | 0.406(0.358-0.459) | 0.375(0.319-0.435) | 0.333(0.224-0.454) | 0.254(0.175-0.348) |
| 17 | With HIV infection | 0.320(0.278-0.367) | 0.297(0.216-0.39) | 0.408(0.274-0.549) | 0.267(0.181-0.372) |
| Cancer | | | | | |
|  | Cancer |  |  |  |  |
| 18 | Diagnosis and primary treatment | 0.303(0.261-0.348) | 0.264(0.176-0.371) | 0.288(0.193-0.399) | 0.174(0.121-0.243) |
| 19 | Metastatic | 0.362(0.317-0.411) | 0.324(0.251-0.407) | 0.451(0.307-0.600) | 0.230(0.158-0.312) |
|  | Terminal phase |  |  |  |  |
| 20 | With medication (for cancers and end-stage kidney or liver disease) | 0.489(0.431-0.547) | 0.515(0.505-0.527) | 0.540(0.377-0.687) | 0.589(0.425-0.743) |
| 21 | Without medication (for cancers and end-stage kidney or liver disease) | 0.412(0.363-0.466) | 0.411(0.369-0.454) | 0.569(0.389-0.727) | 0.537(0.378-0.693) |
| Cardiovascular and circulatory disease | | | | | |
|  | Acute myocardial infarction |  |  |  |  |
| 22 | Days 1-2 | 0.280(0.242-0.324) | 0.261(0.173-0.368) | 0.432(0.288-0.579) | 0.253(0.173-0.353) |
| 23 | Days 3-28 | 0.070(0.058-0.087) | 0.075(0.018-0.202) | 0.074(0.049-0.105) | 0.032(0.019-0.050) |
|  | Angina pectoris |  |  |  |  |
| 24 | Mild | 0.021(0.017-0.026) | 0.037(0.004-0.135) | 0.033(0.020-0.052) | 0.019(0.010-0.033) |
| 25 | Moderate | 0.042(0.034-0.053) | 0.055(0.01-0.173) | 0.080(0.052-0.113) | 0.040(0.024-0.062) |
| 26 | Severe | 0.181(0.154-0.217) | 0.163(0.074-0.297) | 0.167(0.110-0.240) | 0.163(0.111-0.227) |
| 27 | Cardiac conduction disorders and cardiac dysrhythmias | 0.284(0.245-0.327) | 0.256(0.165-0.364) | 0.224(0.151-0.312) | 0.426(0.297-0.561) |
| 28 | Claudication | 0.011(0.009-0.015) | 0.021(0.001-0.095) | 0.014(0.007-0.025) | 0.020(0.011-0.034) |
|  | Heart failure |  |  |  |  |
| 29 | Mild | 0.047(0.038-0.060) | 0.057(0.01-0.175) | 0.041(0.026-0.062) | 0.016(0.008-0.028) |
| 30 | Moderate | 0.095(0.078-0.117) | 0.089(0.024-0.221) | 0.072(0.047-0.103) | 0.041(0.025-0.061) |
| 31 | Severe | 0.187(0.157-0.222) | 0.162(0.073-0.295) | 0.179(0.122-0.251) | 0.240(0.160-0.336) |
|  | Stroke, Long-term consequences |  |  |  |  |
| 32 | Mild | 0.045(0.036-0.055) | 0.053(0.009-0.169) | 0.019(0.010-0.032) | 0.019(0.010-0.033) |
| 33 | Moderate | 0.050(0.040-0.062) | 0.057(0.01-0.175) | 0.070(0.046-0.099) | 0.044(0.027-0.067) |
| 34 | Moderate, plus cognition problems | 0.129(0.107-0.155) | 0.111(0.036-0.247) | 0.316(0.206-0.437) | 0.108(0.074-0.150) |
| 35 | Severe | 0.360(0.315-0.409) | 0.355(0.29-0.423) | 0.552(0.377-0.707) | 0.550(0.386-0.701) |
| 36 | Severe, plus cognition problems | 0.386(0.340-0.437) | 0.400(0.353-0.449) | 0.588(0.411-0.744) | 0.579(0.411-0.732) |
| Diabetes and digestive and genitourinary disease | | | | | |
| 37 | Diabetic neuropathy | 0.104(0.084-0.128) | 0.094(0.027-0.227) | 0.133(0.089-0.187) | 0.098(0.063-0.136) |
| 38 | Chronic kidney disease (stage 4) | 0.083(0.067-0.101) | 0.080(0.02-0.21) | 0.104(0.070-0.147) | 0.106(0.070-0.151) |
|  | End-stage renal disease |  |  |  |  |
| 39 | With kidney transplantation | 0.072(0.058-0.090) | 0.071(0.016-0.197) | 0.024(0.014-0.039) | 0.018(0.010-0.032) |
| 40 | On dialysis | 0.587(0.496-0.678) | 0.672(0.594-0.745) | 0.571(0.398-0.725) | 0.278(0.189-0.382) |
| 41 | Decompensated liver cirrhosis | 0.125(0.103-0.151) | 0.108(0.035-0.244) | 0.178(0.123-0.250) | 0.101(0.067-0.144) |
| 42 | Gastric bleeding | 0.383(0.335-0.433) | 0.390(0.338-0.443) | 0.325(0.209-0.462) | 0.541(0.387-0.690) |
| 43 | Crohn's disease or ulcerative colitis | 0.167(0.139-0.202) | 0.135(0.053-0.271) | 0.231(0.156-0.320) | 0.219(0.152-0.308) |
| 44 | Benign prostatic hypertrophy: symptomatic | 0.087(0.071-0.108) | 0.088(0.023-0.221) | 0.067(0.043-0.097) | 0.096(0.064-0.136) |
| 45 | Urinary incontinence | 0.251(0.215-0.292) | 0.234(0.143-0.351) | 0.139(0.094-0.198) | 0.210(0.143-0.292) |
| 46 | Stress incontinence | 0.030(0.024-0.037) | 0.044(0.006-0.151) | 0.020(0.011-0.035) | 0.014(0.007-0.026) |
| 47 | Impotence | 0.048(0.039-0.061) | 0.058(0.011-0.177) | 0.017(0.009-0.030) | 0.017(0.009-0.030) |
|  | Infertility |  |  |  |  |
| 48 | Primary | 0.016(0.012-0.020) | 0.026(0.002-0.111) | 0.008(0.003-0.015) | 0.009(0.004-0.018) |
| 49 | Secondary | 0.010(0.008-0.014) | 0.018(0.001-0.088) | 0.005(0.002-0.011) | 0.008(0.003-0.016) |
| Chronic respiratory disease | | | | | |
|  | Asthma |  |  |  |  |
| 50 | Controlled | 0.021(0.017-0.027) | 0.032(0.003-0.126) | 0.015(0.007-0.026) | 0.006(0.003-0.013) |
| 51 | Partly controlled | 0.075(0.060-0.093) | 0.081(0.02-0.211) | 0.036(0.022-0.055) | 0.044(0.027-0.065) |
| 52 | Uncontrolled | 0.246(0.211-0.285) | 0.227(0.136-0.346) | 0.133(0.086-0.192) | 0.212(0.145-0.294) |
|  | COPD and other chronic respiratory problems |  |  |  |  |
| 53 | Mild | 0.011(0.009-0.015) | 0.022(0.002-0.101) | 0.019(0.011-0.033) | 0.008(0.003-0.016) |
| 54 | Moderate | 0.211(0.180-0.248) | 0.176(0.086-0.308) | 0.225(0.153-0.310) | 0.232(0.158-0.319) |
| 55 | Severe | 0.316(0.277-0.363) | 0.284(0.200-0.383) | 0.408(0.273-0.556) | 0.299(0.203-0.405) |
| Neurological disorders | | | | | |
|  | Dementia |  |  |  |  |
| 56 | Mild | 0.038(0.031-0.048) | 0.047(0.007-0.157) | 0.069(0.046-0.099) | 0.037(0.022-0.056) |
| 57 | Moderate | 0.191(0.159-0.226) | 0.167(0.077-0.3) | 0.377(0.252-0.508) | 0.382(0.263-0.519) |
| 58 | Severe | 0.302(0.261-0.346) | 0.276(0.191-0.378) | 0.449(0.304-0.595) | 0.511(0.358-0.657) |
| 59 | Migraine | 0.421(0.370-0.478) | 0.416(0.375-0.457) | 0.441(0.294-0.588) | 0.518(0.365-0.668) |
| 60 | Tension-type | 0.173(0.143-0.204) | 0.153(0.065-0.288) | 0.037(0.022-0.057) | 0.109(0.074-0.153) |
| 61 | Medication overuse | 0.226(0.193-0.266) | 0.184(0.093-0.314) | 0.223(0.146-0.313) | 0.185(0.129-0.262) |
|  | Multiple sclerosis |  |  |  |  |
| 62 | Mild | 0.134(0.112-0.163) | 0.129(0.048-0.266) | 0.183(0.124-0.253) | 0.235(0.160-0.324) |
| 63 | Moderate | 0.377(0.330-0.426) | 0.389(0.34-0.442) | 0.463(0.313-0.613) | 0.467(0.325-0.615) |
| 64 | Severe | 0.594(0.499-0.688) | 0.661(0.586-0.731) | 0.719(0.534-0.858) | 0.653(0.483-0.798) |
|  | Epilepsy |  |  |  |  |
| 65 | Severe (seizures once per month or more) | 0.419(0.368-0.473) | 0.462(0.441-0.481) | 0.552(0.375-0.710) | 0.533(0.373-0.686) |
| 66 | Less severe (seizures less than once per month) | 0.383(0.336-0.437) | 0.415(0.376-0.457) | 0.263(0.173-0.367) | 0.396(0.273-0.525) |
|  | Parkinson disease |  |  |  |  |
| 67 | Mild | 0.022(0.018-0.028) | 0.033(0.003-0.129) | 0.010(0.005-0.019) | 0.016(0.008-0.028) |
| 68 | Moderate | 0.350(0.303-0.399) | 0.318(0.241-0.403) | 0.267(0.181-0.372) | 0.193(0.133-0.269) |
| 69 | Severe | 0.451(0.400-0.507) | 0.475(0.458-0.488) | 0.575(0.396-0.730) | 0.527(0.372-0.681) |
| Mental, behavioral, and substance use disorders | | | | | |
|  | Alcohol use disorder |  |  |  |  |
| 70 | Very mild | 0.049(0.040-0.062) | 0.060(0.011-0.18) | 0.123(0.082-0.177) | 0.064(0.040-0.092) |
| 71 | Mild | 0.152(0.127-0.181) | 0.144(0.058-0.28) | 0.235(0.160-0.327) | 0.219(0.151-0.306) |
| 72 | Moderate | 0.234(0.201-0.273) | 0.225(0.133-0.344) | 0.373(0.248-0.508) | 0.312(0.213-0.422) |
| 73 | Severe | 0.315(0.272-0.359) | 0.289(0.202-0.386) | 0.570(0.396-0.732) | 0.413(0.282-0.551) |
|  | Fetal alcohol syndrome |  |  |  |  |
| 74 | Mild | 0.017(0.013-0.022) | 0.028(0.003-0.115) | 0.016(0.008-0.030) | NA |
| 75 | Moderate | 0.117(0.096-0.140) | 0.109(0.036-0.245) | 0.056(0.035-0.083) | NA |
| 76 | Severe | 0.251(0.213-0.291) | 0.201(0.109-0.326) | 0.179(0.119-0.257) | NA |
|  | Cannabis dependence |  |  |  |  |
| 77 | Mild | 0.405(0.357-0.459) | 0.380(0.327-0.438) | 0.039(0.024-0.060) | NA |
| 78 | Mode rate to severe | 0.561(0.483-0.643) | 0.597(0.55-0.645) | 0.266(0.178-0.364) | NA |
|  | Amphetamine dependence |  |  |  |  |
| 79 | Mild | 0.348(0.304-0.396) | 0.318(0.241-0.404) | 0.079(0.051-0.114) | NA |
| 80 | Mode rate to severe | 0.573(0.488-0.658) | 0.622(0.565-0.679) | 0.486(0.329-0.637) | NA |
|  | Cocaine dependence |  |  |  |  |
| 81 | Mild | 0.326(0.283-0.372) | 0.287(0.205-0.385) | 0.116(0.074-0.165) | NA |
| 82 | Mode rate to severe | 0.477(0.421-0.535) | 0.497(0.486-0.506) | 0.479(0.324-0.634) | NA |
|  | Heroin and other opioid dependence |  |  |  |  |
| 83 | Mild | 0.474(0.419-0.532) | 0.494(0.483-0.502) | 0.335(0.221-0.473) | NA |
| 84 | Mode rate to severe | 0.668(0.512-0.784) | 0.752(0.64-0.841) | 0.697(0.510-0.843) | NA |
|  | Anxiety disorders |  |  |  |  |
| 85 | Mild | 0.018(0.014-0.023) | 0.029(0.003-0.118) | 0.030(0.018-0.046) | 0.014(0.007-0.026) |
| 86 | Moderate | 0.132(0.108-0.158) | 0.115(0.040-0.253) | 0.133(0.091-0.186) | 0.108(0.072-0.150) |
| 87 | Severe | 0.538(0.468-0.612) | 0.556(0.528-0.588) | 0.523(0.362-0.677) | 0.376(0.258-0.509) |
|  | Major depressive disorder |  |  |  |  |
| 88 | Mild episode | 0.046(0.037-0.058) | 0.053(0.009-0.168) | 0.145(0.099-0.209) | 0.060(0.038-0.088) |
| 89 | Moderate episode | 0.481(0.424-0.540) | 0.509(0.5-0.52) | 0.396(0.267-0.531) | 0.302(0.204-0.410) |
| 90 | Severe episode | 0.629(0.506-0.735) | 0.699(0.608-0.777) | 0.658(0.477-0.807) | 0.533(0.378-0.680) |
|  | Bipolar disorder |  |  |  |  |
| 91 | Manic episode | 0.479(0.424-0.538) | 0.501(0.492-0.51) | 0.492(0.341-0.646) | 0.321(0.220-0.439) |
| 92 | Residual state | 0.054(0.044-0.068) | 0.060(0.012-0.181) | 0.032(0.018-0.051) | 0.031(0.017-0.048) |
|  | Schizophrenia |  |  |  |  |
| 93 | Acute state | 0.629(0.508-0.739) | 0.711(0.616-0.797) | 0.778(0.606-0.900) | 0.575(0.408-0.733) |
| 94 | Residual state | 0.529(0.463-0.598) | 0.590(0.548-0.633) | 0.588(0.411-0.754) | 0.412(0.283-0.554) |
| 95 | Anorexia nervosa | 0.098(0.080-0.120) | 0.093(0.026-0.226) | 0.224(0.150-0.312) | 0.196(0.137-0.272) |
| 96 | Bulimia nervosa | 0.058(0.047-0.073) | 0.070(0.015-0.195) | 0.223(0.149-0.311) | 0.280(0.191-0.378) |
| 97 | Attention deficit hyperactivity disorder | 0.015(0.012-0.020) | 0.028(0.002-0.116) | 0.045(0.028-0.066) | 0.052(0.032-0.076) |
| 98 | Conduct disorder | 0.273(0.235-0.316) | 0.241(0.149-0.354) | 0.241(0.159-0.341) | 0.243(0.167-0.332) |
| 99 | Borderline intellectual functioning | 0.007(0.005-0.011) | 0.015(0.001-0.078) | 0.011(0.005-0.020) | NA |
|  | Intellectual disability/mental retardation |  |  |  |  |
| 100 | Mild | 0.062(0.050-0.078) | 0.075(0.018-0.202) | 0.043(0.026-0.064) | 0.047(0.030-0.071) |
| 101 | Moderate | 0.095(0.077-0.118) | 0.096(0.028-0.229) | 0.100(0.066-0.142) | 0.074(0.047-0.108) |
| 102 | Severe | 0.119(0.098-0.144) | 0.115(0.039-0.252) | 0.160(0.107-0.226) | 0.122(0.083-0.172) |
| 103 | Profound | 0.263(0.226-0.305) | 0.228(0.136-0.348) | 0.200(0.133-0.283) | 0.230(0.159-0317) |
| Hearing and vision loss | | | | | |
|  | Hearing loss |  |  |  |  |
| 104 | Mild | 0.019(0.016-0.025) | 0.031(0.003-0.122) | 0.010(0.004-0.019) | 0.027(0.015-0.044) |
| 105 | Moderate | 0.067(0.055-0.082) | 0.068(0.014-0.192) | 0.027(0.015-0.042) | 0.038(0.023-0.057) |
| 106 | Severe | 0.288(0.249-0.332) | 0.246(0.156-0.358) | 0.158(0.105-0.227) | 0.208(0.143-0.294) |
| 107 | Profound | 0.245(0.208-0.286) | 0.200(0.109-0.327) | 0.204(0.134-0.288) | 0.241(0.167-0.338) |
| 108 | Complete | 0.166(0.138-0.197) | 0.151(0.065-0.287) | 0.215(0.144-0.307) | 0.300(0.206-0.409) |
| 109 | Moderate (Modified)* | 0.062(0.050-0.078) | 0.068(0.014-0.193) | NA | NA |
| 110 | Severe (Modified)* | 0.266(0.229-0.307) | 0.222(0.131-0.342) | NA | NA |
| 111 | Mild, with ringing | 0.029(0.023-0.036) | 0.042(0.006-0.146) | 0.021(0.012-0.036) | 0.047(0.029-0.069) |
| 112 | Moderate, with ringing | 0.067(0.054-0.083) | 0.076(0.017-0.204) | 0.074(0.049-0.407) | 0.119(0.080-0.166) |
| 113 | Severe, with ringing | 0.262(0.224-0.306) | 0.233(0.140-0.351) | 0.261(0.175-0.360) | 0.280(0.193-0.386) |
| 114 | Profound, with ringing | 0.229(0.197-0.268) | 0.183(0.093-0.313) | 0.277(0.182-0.387) | 0.307(0.214-0.414) |
| 115 | Complete, with ringing | 0.273(0.234-0.314) | 0.236(0.145-0.353) | 0.316(0.212-0.435) | 0.379(0.266-0.514) |
|  | Distance vision |  |  |  |  |
| 116 | Mild impairment | 0.003(0.002-0.008) | 0.009(0.0003-0.057) | 0.003(0.001-0.007) | 0.005(0.002-0.012) |
| 117 | Moderate impairment | 0.021(0.017-0.027) | 0.040(0.005-0.144) | 0.031(0.019-0.049) | 0.051(0.032-0.074) |
| 118 | Severe impairment | 0.246(0.211-0.286) | 0.219(0.127-0.34) | 0.184(0.125-0.258) | 0.378 (0.266-0.514) |
| 119 | Blindness | 0.239(0.203-0.279) | 0.221(0.129-0.341) | 0.187(0.124-0.260) | 0.427 (0.299-0.570) |
| 120 | Monocular impairment | 0.037(0.030-0.048) | 0.052(0.008-0.165) | 0.017(0.009-0.029) | NA |
| 121 | Presbyopia | 0.007(0.005-0.011) | 0.016(0.001-0.081) | 0.011(0.005-0.020) | NA |
| Musculoskeletal disorders | | | | | |
|  | Low back pain |  |  |  |  |
| 122 | Mild | 0.034(0.027-0.043) | 0.046(0.007-0.156) | 0.020(0.011-0.035) | 0.028 (0.016-0.045) |
| 123 | Moderate | 0.126(0.104-0.154) | 0.115(0.039-0.25) | 0.054(0.035-0.079) | 0.069 (0.044-0.100) |
| 124 | Severe, without leg pain | 0.181(0.152-0.214) | 0.164(0.076-0.298) | 0.272(0.182-0.373) | NA |
| 125 | Severe, with leg pain | 0.210(0.179-0.246) | 0.180(0.089-0.311) | 0.325(0.219-0.446) | NA |
| 126 | Most severe, without leg pain | 0.221(0.187-0.259) | 0.180(0.09-0.312) | 0.372(0.250-0.506) | NA |
| 127 | Most severe, with leg pain | 0.224(0.190-0.262) | 0.188(0.095-0.317) | 0.384(0.256-0.518) | NA |
|  | Neck pain |  |  |  |  |
| 128 | Mild | 0.033(0.026-0.042) | 0.047(0.007-0.159) | 0.053(0.034-0.078) | 0.039 (0.023-0.058) |
| 129 | Moderate | 0.061(0.050-0.075) | 0.070(0.015-0.196) | 0.114(0.075-0.162) | 0.063 (0.041-0.091) |
| 130 | Severe | 0.132(0.109-0.159) | 0.126(0.045-0.263) | 0.229(0.153-0.317) | 0.169 (0.115-0.236) |
| 131 | Most severe | 0.107(0.088-0.130) | 0.106(0.033-0.242) | 0.304(0.202-0.415) | 0.144 (0.099-0.200) |
|  | Musculoskeletal problems |  |  |  |  |
| 132 | Legs, mild | 0.025(0.020-0.032) | 0.043(0.006-0.149) | 0.023(0.013-0.037) | 0.091 (0.060-0.129) |
| 133 | Legs, moderate | 0.120(0.099-0.144) | 0.122(0.043-0.259) | 0.079(0.054-0.110) | 0.142 (0.098-0.196) |
| 134 | Legs, severe | 0.169(0.140-0.200) | 0.162(0.073-0.294) | 0.165(0.112-0.232) | 0.327 (0.223-0.438) |
| 135 | Arms, mild | 0.038(0.031-0.047) | 0.047(0.007-0.157) | 0.028(0.017-0.045) | 0.039 (0.023-0.058) |
| 136 | Arms, moderate | 0.125(0.102-0.151) | 0.123(0.044-0.26) | 0.117(0.080-0.163) | 0.223 (0.153-0.307) |
| 137 | Generalized, moderate | 0.151(0.125-0.181) | 0.138(0.055-0.275) | 0.317(0.216-0.440) | 0.255 (0.177-0.352) |
| 138 | Generalized, severe | 0.332(0.287-0.379) | 0.306(0.228-0.396) | 0.581(0.403-0.739) | 0.420(0.293-0.560) |
| 139 | Gout: acute | 0.231(0.198-0.269) | 0.212(0.119-0.335) | 0.295(0.196-0.409) | 0.322(0.221-0.436) |
| Injury | | | | | |
| 140 | Amputation, One arm: long term, without treatment | 0.107(0.088-0.131) | 0.117(0.04-0.253) | 0.118(0.079-0.167) | 0.261(0.182-0.357) |
| 141 | Concussion | 0.095(0.077-0.115) | 0.083(0.021-0.213) | 0.110(0.074-0.158) | 0.170(0.112-0.244) |
| 142 | Spinal cord lesion, Below neck： treated | 0.352(0.308-0.399) | 0.359(0.297-0.425) | 0.296(0.198-0.414) | 0.388 (0.270-0.524) |
| Other | | | | | |
|  | Abdominopelvic problem |  |  |  |  |
| 143 | Mild | 0.031(0.025-0.039) | 0.039(0.005-0.143) | 0.011(0.005-0.021) | 0.029 (0.016-0.046) |
| 144 | Moderate | 0.204(0.172-0.240) | 0.175(0.084-0.307) | 0.114(0.078-0.159) | 0.392 (0.270-0.524) |
| 145 | Severe | 0.427(0.376-0.480) | 0.420(0.382-0.459) | 0.324(0.220-0.442) | 0.339 (0.235-0.458) |
|  | Anemia |  |  |  |  |
| 146 | Mild | 0.003(0.002-0.008) | 0.009(0.0003-0.057) | 0.004(0.001-0.008) | 0.004 (0.001-0.009) |
| 147 | Moderate | 0.104(0.083-0.126) | 0.094(0.026-0.226) | 0.052(0.034-0.076) | 0.064 (0.040-0.092) |
| 148 | Severe | 0.219(0.186-0.256) | 0.179(0.088-0.31) | 0.149(0.101-0.209) | 0.040 (0.024-0.061) |
| 149 | Periodontitis | 0.011(0.008-0.015) | 0.019(0.001-0.091) | 0.007(0.003-0.014) | 0.008 (0.003-0.015) |
| 150 | Dental caries： symptomatic | 0.009(0.007-0.013) | 0.018(0.001-0.087) | 0.010(0.005-0.019) | 0.035 (0.021-0.053) |
| 151 | Severe tooth loss | 0.033(0.026-0.042) | 0.046(0.007-0.156) | 0.067(0.045-0.095) | 0.082 (0.053-0.115) |
|  | Disfigurement |  |  |  |  |
| 152 | Level 1 | 0.033(0.027-0.041) | 0.048(0.007-0.159) | 0.011(0.005-0.021) | 0.043 (0.026-0.063) |
| 153 | Level 2 | 0.168(0.140-0.202) | 0.154(0.067-0.289) | 0.067(0.044-0.096) | 0.123 (0.083-0.171) |
| 154 | Level 3 | 0.520(0.456-0.587) | 0.572(0.537-0.608) | 0.405(0.275-0.546) | 0.512 (0.362-0.670) |
| 155 | Level 1, with itch or pain | 0.048(0.039-0.061) | 0.063(0.013-0.186) | 0.027(0.015-0.042) | NA |
| 156 | Level 2, with itch or pain | 0.225(0.191-0.262) | 0.192(0.1-0.321) | 0.188(0.125-0.267) | NA |
| 157 | Level 3, with itch or pain | 0.566(0.485-0.650) | 0.625(0.567-0.683) | 0.576(0.401-0.731) | NA |
|  | Generic uncomplicated disease |  |  |  |  |
| 158 | Worry and daily medication | 0.032(0.026-0.041) | 0.045(0.006-0.153) | 0.049(0.031-0.072) | 0.016 (0.008-0.028) |
| 159 | Anxiety about diagnosis | 0.009(0.007-0.013) | 0.018(0.001-0.087) | 0.012(0.006-0.023) | 0.008 (0.003-0.015) |
| 160 | Kwashiorkor | 0.083(0.067-0.104) | 0.079(0.019-0.207) | 0.051(0.031-0.079) | NA |
| 161 | Severe wasting | 0.198(0.168-0.234) | 0.154(0.067-0.287) | 0.128(0.082-0.183) | 0.086 (0.056-0.124) |
| 162 | Speech problems | 0.028(0.023-0.036) | 0.041(0.005-0.146) | 0.051(0.032-0.078) | 0.065(0.041-0.094) |
|  | Motor impairment |  |  |  |  |
| 163 | Mild | 0.018(0.015-0.024) | 0.029(0.003-0.119) | 0.010(0.005-0.019) | 0.009(0.004-0.016) |
| 164 | Moderate | 0.033(0.027-0.041) | 0.044(0.006-0.151) | 0.061(0.040-0.089) | 0.045(0.028-0.065) |
| 165 | Severe | 0.290(0.251-0.332) | 0.262(0.174-0.37) | 0.402(0.268-0.545) | 0.294(0.202-0.396) |
|  | Motor plus cognitive impairments |  |  |  |  |
| 166 | Mild | 0.021(0.017-0.027) | 0.036(0.004-0.135) | 0.031(0.018-0.050) | 0.023(0.013-0.038) |
| 167 | Moderate | 0.067(0.055-0.082) | 0.075(0.017-0.202) | 0.203(0.134-0.290) | 0.106(0.069-0.150) |
| 168 | Severe | 0.285(0.246-0.331) | 0.271(0.184-0.373) | 0.542(0.374-0.702) | 0.457(0.318-0.606) |
| 169 | Rectovaginal fistula | 0.338(0.296-0.386) | 0.340(0.271-0.416) | 0.501(0.339-0.657) | NA |
| 170 | Vesicovaginal fistula | 0.255(0.217-0.297) | 0.229(0.136-0.347) | 0.342(0.227-0.478) | NA |
| 171 | Thrombocytopenic purpura | 0.141(0.116-0.170) | 0.131(0.05-0.268) | 0.159(0.106-0.226) | 0.110(0.073-0.154) |
| 172 | Hypothyroidism | 0.020(0.016-0.025) | 0.030(0.003-0.12) | 0.019(0.010-0.032) | 0.012(0.005-0.023) |
| 173 | Hyperthyroidism | 0.139(0.115-0.168) | 0.117(0.04-0.253) | 0.145(0.096-0.202) | 0.103(0.069-0.146) |
| 174 | Vertigo | 0.050(0.041-0.063) | 0.065(0.013-0.186) | 0.113(0.074-0.158) | 0.102(0.069-0.145) |
| 175 | Often has a burning sensation in the back of the chest after eating | 0.095(0.077-0.116) | 0.088(0.024-0.219) | NA | 0.046(0.029-0.069) |
| 176 | Stress incontinence | 0.028(0.023-0.036) | 0.046(0.006-0.155) | NA | NA |
| 177 | Neck pain, moderate | 0.102(0.084-0.125) | 0.103(0.032-0.24) | NA | NA |
| 178 | Allergic rhinitis (hay fever) | 0.006(0.004-0.010) | 0.015(0.001-0.076) | NA | 0.009(0.004-0.016) |
| 179 | Heart burn & reflux “GERD” | 0.099(0.081-0.122) | 0.092(0.025-0.225) | NA | 0.046(0.029-0.069) |
| 180 | Constipation | 0.056(0.046-0.070) | 0.061(0.012-0.181) | NA | 0.048(0.030-0.070) |
| 181 | Haemorrhoids | 0.092(0.074-0.112) | 0.091(0.025-0.224) | NA | NA |
| 182 | Somatoform disorder | 0.112(0.093-0.136) | 0.097(0.028-0.231) | NA | 0.060(0.038-0.086) |
| 183 | Insomnia | 0.032(0.026-0.040) | 0.040(0.005-0.144) | NA | 0.036(0.022-0.055) |
| 184 | Sleep apnoea | 0.082(0.066-0.102) | 0.080(0.02-0.209) | NA | 0.024(0.014-0.040) |
| 185 | Hypothyroidism | 0.030(0.025-0.038) | 0.042(0.006-0.146) | NA | NA |
| 186 | Borderline personality disorder | 0.255(0.220-0.298) | 0.196(0.103-0.322) | NA | 0.132(0.091-0.186) |
| 187 | Harmful alcohol use | 0.100(0.082-0.122) | 0.098(0.029-0.233) | NA | NA |
| 188 | Hyperthyroidism | 0.073(0.059-0.091) | 0.073(0.016-0.198) | NA | NA |
| 189 | Vaginal discharge | 0.016(0.013-0.020) | 0.028(0.002-0.115) | NA | 0.096(0.062-0.135) |
| 190 | Dyspareunia | 0.034(0.028-0.044) | 0.044(0.006-0.151) | NA | NA |
| 191 | Trigeminal neuralgia | 0.090(0.073-0.111) | 0.088(0.024-0.220) | NA | 0.128(0.086-0.181) |
| 192 | Anal fissure/abcess/fistula | 0.056(0.046-0.070) | 0.069(0.015-0.194) | NA | NA |
| 193 | Varicose veins | 0.020(0.016-0.026) | 0.039(0.005-0.141) | NA | 0.018(0.010-0.031) |
| 194 | Carpal tunnel syndrome | 0.017(0.014-0.022) | 0.028(0.003-0.117) | NA | 0.020(0.011-0.034) |
| 195 | Osteomyelitis | 0.105(0.086-0.128) | 0.107(0.034-0.242) | NA | NA |
| 196 | Shoulder lesions | 0.019(0.015-0.024) | 0.031(0.003-0.123) | NA | NA |
| 197 | Irritable bowel syndrome | 0.036(0.029-0.044) | 0.045(0.006-0.151) | NA | 0.039(0.024-0.059) |
| 198 | Thrombocytopenic purpura | 0.160(0.134-0.193) | 0.141(0.056-0.277) | NA | NA |
| 199 | Lymphogranuloma Venereum -local infection | 0.098(0.081-0.120) | 0.104(0.032-0.238) | NA | NA |
| 200 | Intensive care unit admission | 0.601(0.497-0.699) | 0.669(0.589-0.741) | NA | 0.675(0.506-0.822) |
| 201 | Invasive device/drain | 0.191(0.161-0.227) | 0.175(0.084-0.307) | NA | 0.512(0.362-0.664) |
|  | Encephalopathy |  |  |  |  |
| 202 | Moderate | 0.212(0.181-0.251) | 0.175(0.085-0.306) | NA | NA |
| 203 | Severe | 0.309(0.268-0.354) | 0.285(0.200-0.383) | NA | NA |
|  | Subacute sclerosing panencephalitis |  |  |  |  |
| 204 | Phase 1 | 0.051(0.041-0.063) | 0.056(0.010-0.173) | NA | NA |
| 205 | Phase 2 | 0.189(0.160-0.225) | 0.151(0.065-0.286) | NA | NA |
| 206 | Phase 3 | - | - | NA | NA |

NA: Not available

# Supplementary Table S4 **Comparison of the health states between Anhui Province and other studies**

| Category of disease | Numbers of health states | | | | |  | Numbers of health states matched with Anhui | | | |  | Numbers of health states with more than 3 times DW compared with Anhui's DW | | | |
| --- | --- | --- | --- | --- | --- | --- | --- | --- | --- | --- | --- | --- | --- | --- | --- |
|  | Anhui | GBD[12] | China[17] | Janpan[16] | Korea[13] |  | GBD[12] | China[17] | Janpan[16] | Korea[13] |  | GBD | China | Janpan | Korea |
| Infectious disease | 17 | 17 | 17 | 15 | 17 |  | 17 | 17 | 15 | 15 |  | 3 | 0 | 1 | 6 |
| Cancer | 4 | 6 | 4 | 7 | 6 |  | 4 | 4 | 4 | 4 |  | 0 | 0 | 0 | 0 |
| Cardiovascular and circulatory disease | 15 | 15 | 15 | 15 | 15 |  | 15 | 15 | 15 | 15 |  | 0 | 0 | 0 | 5 |
| Diabetes and digestive and genitourinary disease | 13 | 14 | 13 | 14 | 13 |  | 13 | 13 | 13 | 12 |  | 1 | 0 | 1 | 3 |
| Chronic respiratory disease | 6 | 6 | 6 | 6 | 6 |  | 6 | 6 | 6 | 6 |  | 0 | 0 | 1 | 3 |
| Neurological disorders | 14 | 14 | 14 | 14 | 15 |  | 14 | 14 | 14 | 13 |  | 1 | 0 | 0 | 2 |
| Mental, behavioral, and substance use disorders | 34 | 36 | 34 | 27 | 30 |  | 34 | 34 | 22 | 28 |  | 5 | 0 | 2 | 5 |
| Hearing and vision loss | 18 | 16 | 18 | 15 | 15 |  | 16 | 18 | 14 | 14 |  | 0 | 1 | 0 | 2 |
| Musculoskeletal disorders | 18 | 18 | 18 | 18 | 59 |  | 18 | 18 | 14 | 8 |  | 0 | 0 | 1 | 3 |
| Injury | 3 | 60 | 3 | 58 | 15 |  | 3 | 3 | 3 | 2 |  | 0 | 0 | 0 | 1 |
| Other | 64 | 33 | 64 | 42 | 65 |  | 32 | 64 | 41 | 26 |  | 2 | 1 | 4 | 10 |
| Total | 206 | 235 | 206 | 231 | 256 |  | 172 | 206 | 161 | 143 |  | 12 | 2 | 10 | 40 |

.**
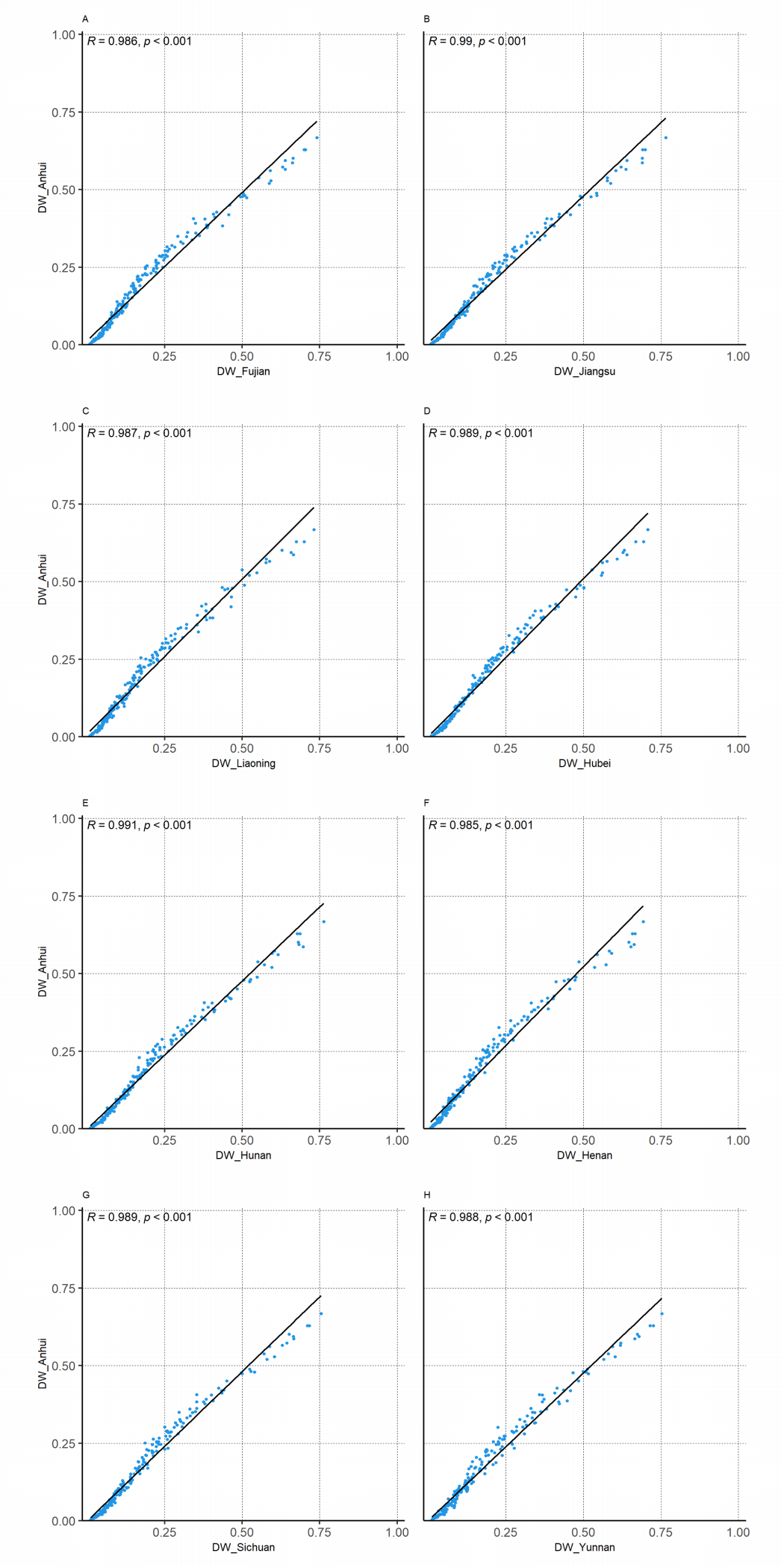
**

# **Supplementary Figure SF1 Correlation between the DWs of Anhui province and those of other provinces in China^17^**

#
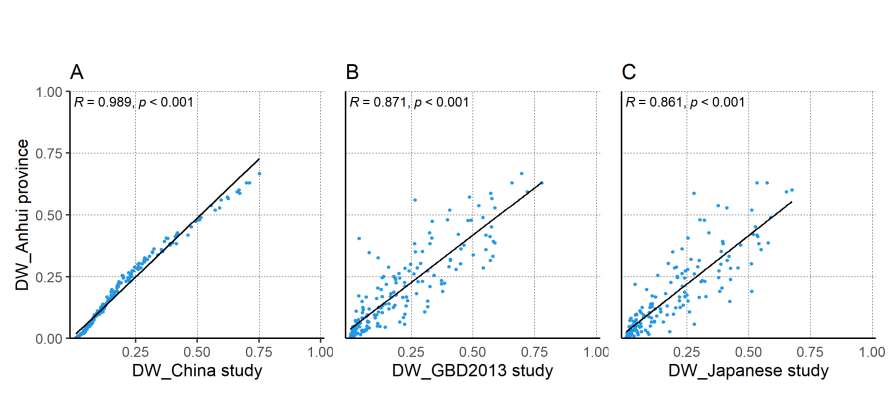
**Supplementary Figure SF2 Correlation between the DWs of Anhui province and those of GBD and Asian countries**


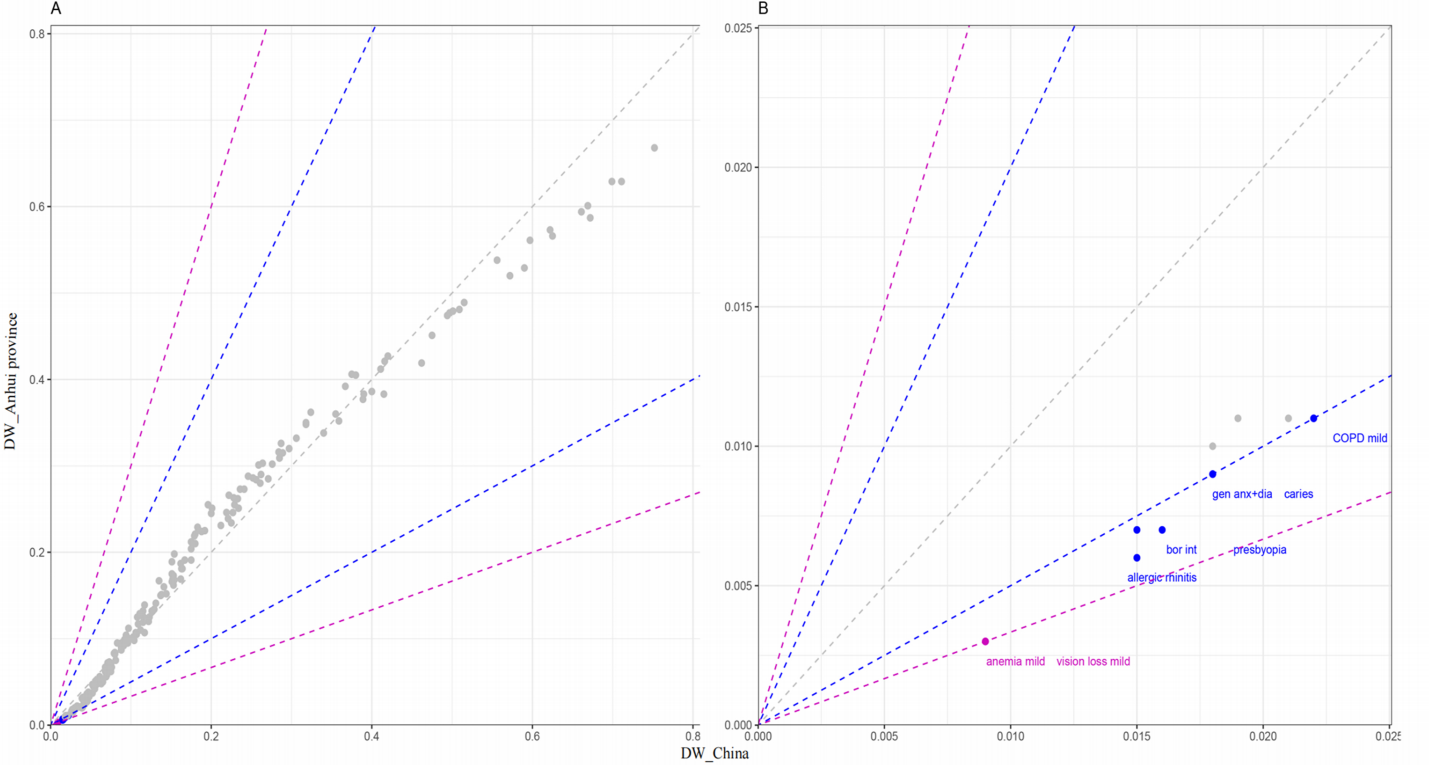


**Supplementary Figure SF3 Comparison of the health states with a difference of more than 2 or 3 times between Anhui province and China^17^** The grey dotted diagonal line indicated the equivalence between DW of Anhui Province and other study, and the blue and purple dashed lines indicated a difference of 2 and 3 times between the DW of Anhui and other study respectively.


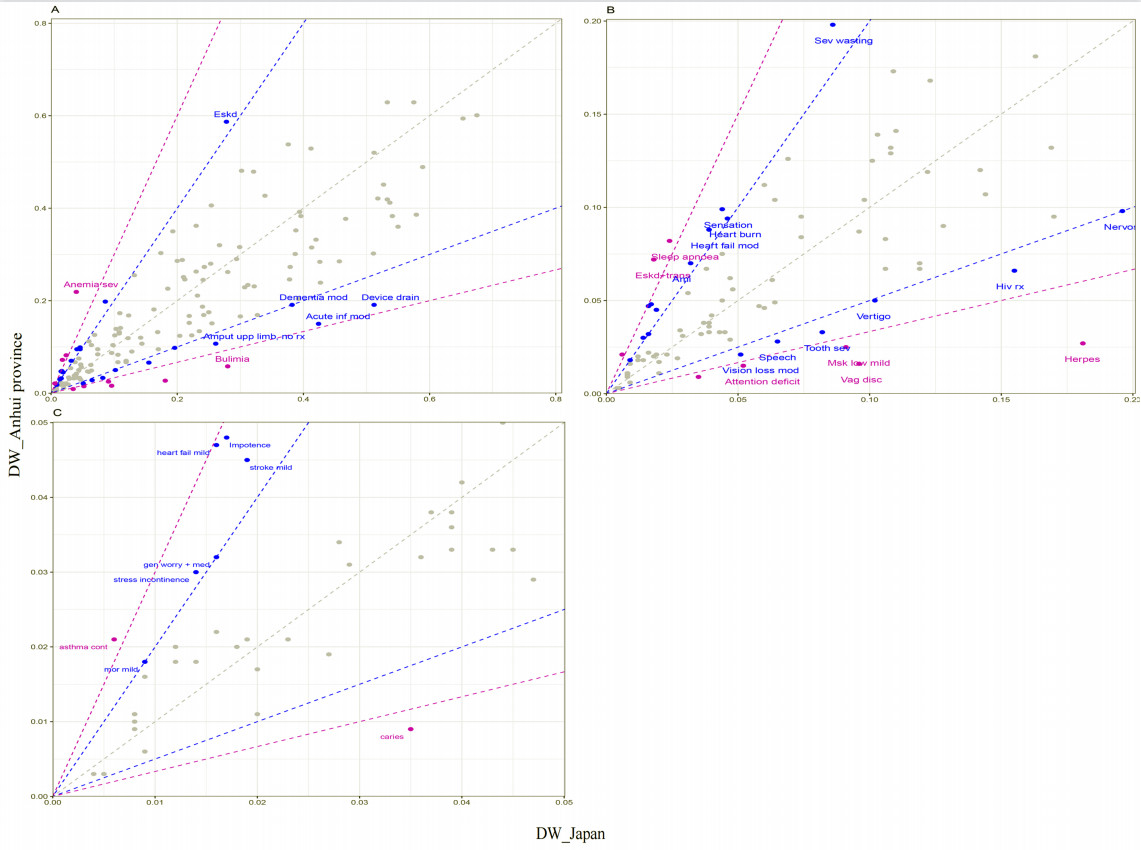


**Supplementary Figure SF4 Comparison of the health states with a difference of more than 2 or 3 times between Anhui province and Japan^16^** The grey dotted diagonal line indicated the equivalence between DW of Anhui Province and other study, and the blue and purple dashed lines indicated a difference of 2 and 3 times between the DW of Anhui and other study respectively.


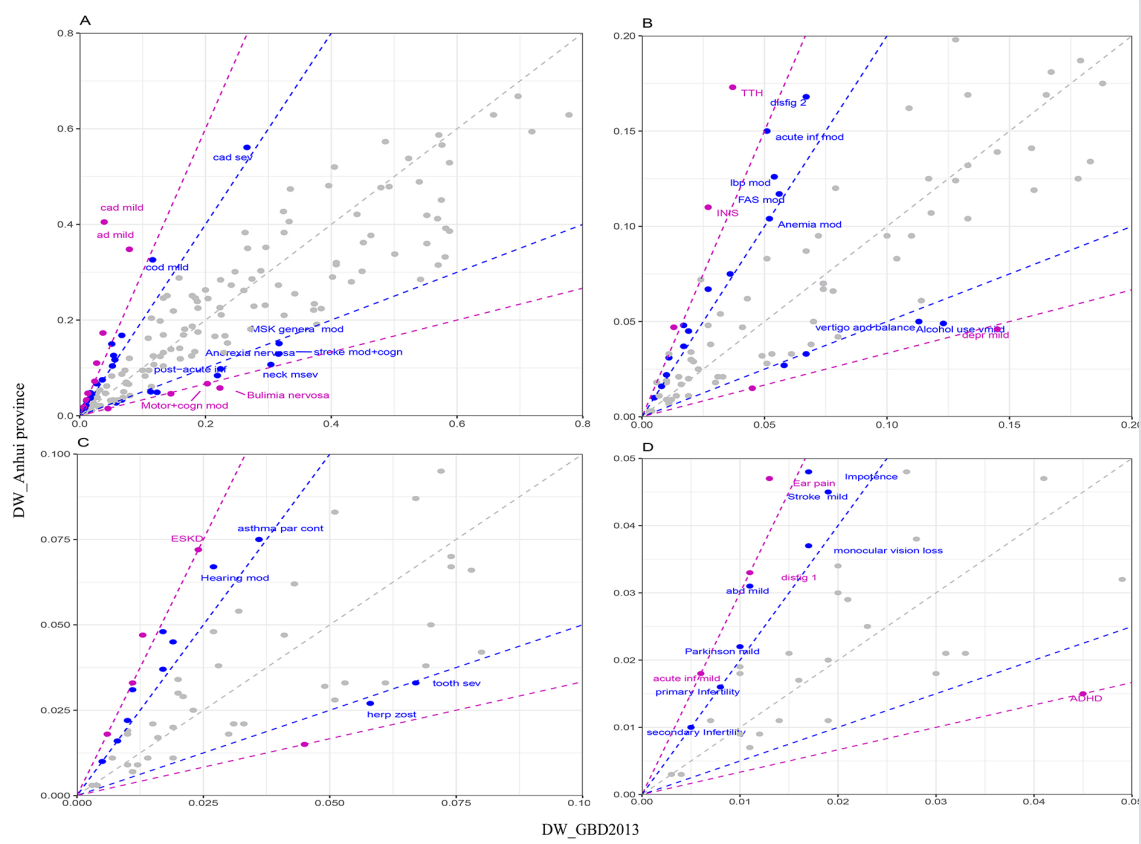


**Supplementary Figure SF5 Comparison of the health states with a difference of more than 2 or 3 times between Anhui province and GBD2013^12^** The grey dotted diagonal line indicated the equivalence between DW of Anhui Province and other study, and the blue and purple dashed lines indicated a difference of 2 and 3 times between the DW of Anhui and other study respectively.
